# A Systematic Review of the Impact of Viral Respiratory Epidemics on Mental Health: An Implication on the Coronavirus Disease 2019 Pandemic

**DOI:** 10.3389/fpsyt.2020.565098

**Published:** 2020-11-23

**Authors:** Yang Luo, Cher Rui Chua, Zhonghui Xiong, Roger C. Ho, Cyrus S. H. Ho

**Affiliations:** ^1^Department of Psychological Medicine, Yong Loo Lin School of Medicine, National University of Singapore, Singapore, Singapore; ^2^Institute for Health Innovation and Technology (iHealthtech), National University of Singapore, Singapore, Singapore; ^3^Department of Psychological Medicine, National University Health System, Singapore, Singapore

**Keywords:** epidemics, coronavirus, COVID-19, SARS, MERS, influenza, mental health

## Abstract

**Background:** The twenty-first century viral respiratory epidemics have taught us valuable lessons. Our systematic review examined the impact of these epidemics, including coronavirus disease 2019 (COVID-19), on mental health among different population groups, drawing on their insights for recommendations for the current COVID-19 pandemic.

**Methods:** Searches were performed on PubMed, Embase, PsycINFO, Web of Science, Scopus, CINAHL, and Cochrane on April 4, 2020. Studies that had undefined mental health outcomes or did not use a validated scale for measure were excluded. Quality assessment was carried out *via* the Newcastle–Ottawa Scale.

**Results:** We included 95 studies, most of which were conducted in Hong Kong (31.6%) and China (21.4%). A total of 30 (30.9%) studies are on the general public, 41 (42.2%) on healthcare workers, and 26 (26.6%) on patients and quarantined individuals. Furthermore, 36 (37.1%) of the studies are of high quality, 48 (49.5%) are of moderate quality, and 13 (13.4%) are of low quality. The most significant mental health outcomes reported include anxiety, depression, and post-traumatic stress disorder symptoms. The subgroups identified to have a higher risk of psychiatric symptoms among the general public include females, the elderly, individuals with chronic illness, migrant workers, and students. Long-term mental health impact was reported in some healthcare workers and epidemic patients, even up to 3 years in the former. Interestingly, when compared to non-quarantined groups, quarantine was not significantly associated with worse mental health outcomes.

**Conclusion:** Important implications for the COVID-19 pandemic were highlighted. Respiratory epidemics pose a significant psychological morbidity onto many population groups. Psychological support for vulnerable groups, including healthcare workers and patients, should be implemented to prevent them from spiraling into clinical psychiatric conditions.

## Introduction

Respiratory epidemics erupted around the world at an unprecedented level in recent years. In 2002, severe acute respiratory syndrome coronavirus (SARS-CoV) resulted in an epidemic involving 26 countries and more than 8,000 people (1). This was soon followed by the influenza A/H1N1 pandemic, the Middle East Respiratory Syndrome (MERS) epidemic, and the influenza A/H7N9 epidemic. As the world becomes increasingly globalized, the spread of highly contagious viruses has never been wider. From December 31, 2019 until May 20, 2020, coronavirus disease 2019 (COVID-19) has infected 4,761,559 people and caused 317,529 deaths (2).

COVID-19 has produced a substantial impact among many groups of people. Amidst the high unemployment rates in this epidemic, a mental health crisis has been brewing, which confers significant psychological morbidity onto vulnerable individuals (3–5). Healthcare workers face an overwhelming patient load and a high risk of infection (6). In the SARS epidemic, quarantined patients faced social isolation and activity restriction (7). Patients who were impacted with the novel respiratory illness had to face the risk of mortality and long-term functional impairment (8).

While many articles addressing the various treatment options and clinical outcomes of patients during these outbreaks emerged, we must not overlook the mental health status of different population groups. Individuals who suffer from psychiatric disorders during and after epidemics confer a less-established medical burden on society that is worth exploring. A well-presented systematic review and meta-analysis, studying the prevalence of psychiatric conditions among healthcare workers during the current COVID-19 pandemic, was conducted (9). A high proportion of healthcare workers experienced symptoms of depression, anxiety, and insomnia. In preventing further deterioration of mental health, timely, and focused interventions should be instituted. Building onto their knowledge, we find value in exploring past epidemics and including a wider scope of coverage to include other population groups.

In this systematic review, we explore the relationship between viral respiratory epidemics in the 21st century and their impact on mental health in populations around the world—particularly the general public, healthcare workers and students, patients of the epidemics, and quarantined individuals. These epidemics, selected due to their common mode of transmission *via* respiratory droplets, include H1N1, H7N9, SARS, MERS, and COVID-19. In conducting this study, we hope to draw on the insights from the included studies and provide recommendations for the current COVID-19 pandemic.

## Methodology

### Search Strategies

This study is in accordance with the Preferred Reporting Items for Systematic Reviews and Meta-Analyses (PRISMA) guidelines (10). A search was conducted on April 4, 2020 on PubMed (2,333), Embase (3,011), PsycINFO (440), Web of Science (4,938), Scopus (3,317), CINAHL (722), and Cochrane (506). A total of 15,267 articles from January 2000 to April 2020 were identified. We used a combination of controlled vocabulary, where appropriate, and free-text terms relating to SARS, MERS-CoV, COVID-19, influenza outbreak, and psychiatric conditions (see Appendix A).

### Inclusion and Exclusion Criteria

Two researchers (YL and CRC) independently screened the titles and the abstracts and assessed the full-text articles to select those that met the criteria. In the case of unresolved disputes between the two researchers, a third researcher (ZX) was involved. We included peer-reviewed observational/experimental studies examining the impact of SARS, MERS-CoV, influenza A/H1N1 and influenza A/H7N9, and COVID-19 on mental health outcomes. The population groups that we included are the general public, healthcare workers, healthcare students, patients of the viral respiratory epidemics, and quarantined individuals. We excluded outbreaks which occurred before year 2000, narrative reviews, systematic reviews, meeting or conference abstracts, commentaries, case reports, protocols, articles which reported unclear outcomes, outcomes not determined by validated scales, and full-text articles not in English.

### Data Extraction

Data were extracted independently into a pre-specified data extraction form and cross-checked by two researchers (YL and CRC). As the data were unsuitable for statistical pooling or meta-analysis, a narrative synthesis was carried out. In our review, long-term mental health outcomes were identified based on a cutoff of 6 months after the epidemics. Data were analyzed separately into subgroups.

### Quality Assessment

Two researchers (YL and CRC) conducted the scoring independently, and discrepancies were resolved by a third researcher (ZX). Quality assessment was carried out using the Newcastle–Ottawa Scale (NOS) for case–control and cohort studies (11). Stars (^*^) are awarded based on the three categories assessed: selection, comparability, and exposure. The maximum number of stars is nine. An adapted NOS by Herzog was used for cross-sectional studies (12). The maximum number of stars is 10. The higher the number of stars that each paper received, the better the research quality. In terms of quality, seven stars or higher is considered high quality, five to six stars as moderate quality, and four stars and below as low quality.

## Results

### Search Results and Study Characteristics

**Figure 1:**
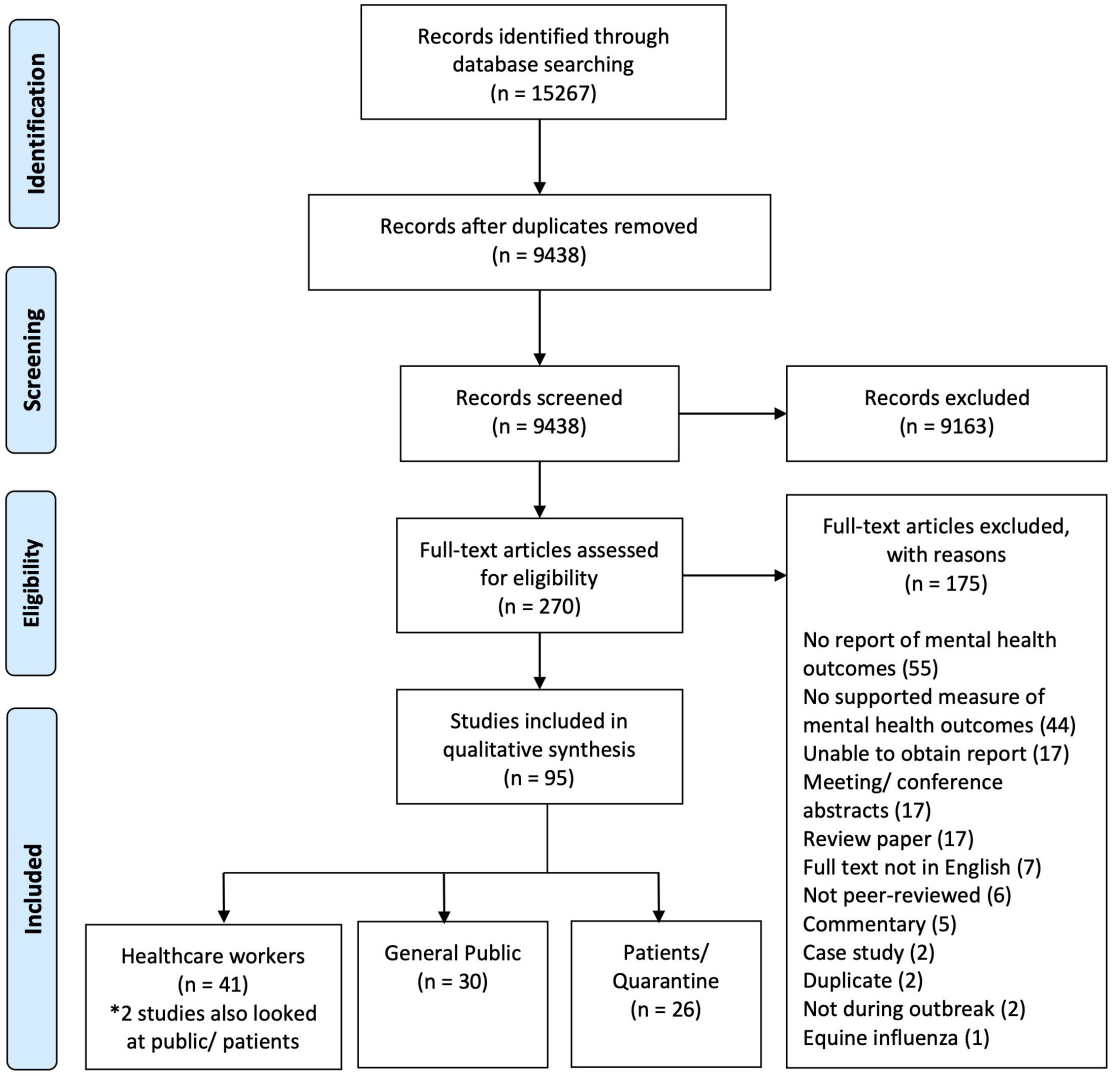
Preferred reporting items for systematic reviews and meta-analyses flow diagram.

We identified 270 potential articles and excluded 175 papers after examination of full text (see Figure 1). A total of 95 papers were included. These papers were divided into three population subgroups, namely, the general public (*n* = 30, 30.9%), healthcare workers (*n* = 41, 42.2%), and patients and quarantined individuals of respiratory epidemics (*n* = 26, 26.6%). The included studies were carried out in 13 regions. These regions included Hong Kong (*n* = 31), China (*n* = 21), Taiwan (*n* = 10), Singapore (*n* = 9), South Korea (*n* = 9), Canada (*n* = 8), Saudi Arabia (*n* = 2), United States (*n* = 2), and others (*n* = 5). The epidemics included are SARS (*n* = 59), influenza (*n* = 14), MERS-CoV (*n* = 12), and COVID-19 (*n* = 10). Of all the mental health outcomes explored, post-traumatic stress disorder (PTSD; *n* = 43), anxiety (*n* = 42), and depression (*n* = 34) were the most prevalent. In terms of timing of study, 52 studies were done during the epidemic, 40 done after, and 5 done before and after. Details of the study characteristics are provided in Table 1.

**Table 1:** Overall study characteristics.

**Characteristics**	***N* (%)**
**Population (*****n*** **=** **97)**	
Healthcare workers	41 (42.2)
General public	30 (30.9)
Patients/quarantined individuals	26 (26.6)
**Outbreaks (*****n*** **=** **95)**	
SARS	59 (62.1)
Influenza	14 (14.7)
MERS-CoV	12 (12.6)
COVID-19	10 (10.5)
**Mental health outcomes (*****n*** **=** **156)**	
Post-traumatic stress disorder	43 (27.6)
Anxiety	42 (26.9)
Depression	34 (21.8)
Others	37 (23.7)
**Countries/regions (*****n*** **=** **98)**	
Hong Kong	31 (31.6)
China	21 (21.4)
Taiwan	10 (10.2)
Singapore	10 (10.2)
South Korea	9 (9.2)
Canada	8 (8.2)
Saudi Arabia	2 (2.0)
United States	2 (2.0)
Others (Greece, India, Japan, Mexico, United Kingdom)	5 (5.1)
*Study period (n = 97)*	
During outbreak	52 (53.6)
After outbreak	40 (41.2)
Both during and after outbreak	5 (5.2)
*Study design (n = 97)*	
Cross-sectional	75 (77.3)
Cohort study	18 (18.6)
Case–control	4

Results specific to each population group will be analyzed in the respective sections. A summary of identified studies can be found in Appendix B. For COVID-19-specific articles, a separate summary can be found in Table 2.

**Table 2:** Summary of the study characteristics for COVID-19 articles.

**References**	**Country**	**Subgroup**	**Reported outcome**	**Timeframe**	**Study design**	**Data collection**	**Sample size**	**Scale used**	**Results**
Li et al. (13)	China	General public, HCW	Vicarious traumatization	During	Cross-sectional	Self-reported questionnaire	214	Vicarious trauma scale	The general public and medical staff suffer from vicarious traumatization. However, the vicarious traumatization of non-front-line medical staff is more serious than that of front-line medical staff.
Liu et al. (14)	China	General public	PTSD	During	Cross-sectional	Self-reported questionnaire	285	PTSD checklist for DSM-5 (PCL-5), Pittsburgh Sleep Quality Index (PSQI)	2019-Cov pandemics have a high prevalence of post-traumatic stress symptoms (PTSS) in the hardest-hit areas in China of 7%. Most importantly, PTSS sub-symptoms, including re-experiencing, negative alterations in cognition or mood, and hyper-arousal are more common in females than males. Better sleep quality and unfragmented sleep patterns are associated with lower PTSS prevalence.
Qiu et al. (15)	China, Hong Kong, Taiwan	General public	Psychological distress	During	Cross-sectional	Online questionnaire	52,730	COVID-19 Peritraumatic Distress Index (CPDI)	Multinomial logistic regression analyses showed that one's CPDI score was associated with female gender, higher education, migrant workers and staying in the middle region of China (most affected by epidemic). Lower psychological distress levels are associated with male gender, availability of local medical resources, efficiency of the regional public health system, and prevention and control measures taken against the epidemic situation, age under 18 years.
Wang et al. (16)	China	General public	Depression, anxiety, PTSD	During	Cross-sectional	Online questionnaire	1,210	Impact of Event Scale-Revised (IES-R), Depression Anxiety Stress Scale (DASS)	Higher IES-R and DASS scores are associated with female gender, student status, specific physical symptoms, and no confidence in their own doctor's ability to diagnose or recognize COVID-19. Higher IES-R scores are associated with high levels of concern about other family members getting COVID-19 and dissatisfaction with the amount of health information available about COVID-19. Higher DASS depression subscale scores are associated with male gender, uneducated status and breathing difficulty. Higher DASS anxiety subscale scores are associated with male gender, clinic consultations and hospitalizations, contact with an individual with suspected COVID-19 or infected materials, breathing difficulty and high levels of concern about other family members getting COVID-19. Higher DASS stress subscale scores are associated with male gender, a low perceived likelihood of surviving COVID-19 if infected, high levels of concern about other family members getting COVID-19 and dissatisfaction with the amount of health information available about COVID-19. Lower IES-R and DASS scores are associated with specific up-to-date and accurate health information and particular precautionary measures. Lower IES-R scores are associated with male gender. Lower DASS depression subscale scores are associated with additional information on availability and effectiveness of medicines/vaccines. Lower DASS anxiety subscale scores are associated with low perceived likelihood of contracting COVID-19, regular updates for the latest information and additional information on the availability and effectiveness of medicines/vaccines. Lower DASS stress subscale scores are associated with low perceived likelihood of contracting COVID-19 and the information on the increase in the number of recovered individuals.
Wang et al. (17)	China	General public	Depression, anxiety	During	Cross-sectional	Online questionnaire	600	Self-Rating Anxiety Scale (SAS), Self-Rating Depression Scale (SDS)	SAS and SDS standard scores showed a significant positive correlation. High risk in female gender, 40 and below age group, those with a master's degree or above (compared to those with a bachelor's degree), professionals (compared to industrial service workers and other staff).
Lai et al. (18)	China	Healthcare workers	PTSD, anxiety	During	Cross-sectional	Self-reported questionnaire	1,257	Patient Health Questionnaire-9 (PHQ-9)Insomnia Severity Index (ISI-7)General Anxiety Disorder-7 criteria (GAD-7)	More severe symptoms in all areas in these populations: nurses, women, and frontline workers. Significantly higher symptoms of depression (OR = 1.52, 95% CI = 1.11–2.09, *p* = 0.01), anxiety (OR = 1.57, 95% CI = 1.22-2.02, *p* < 0.001), insomnia (OR = 2.97, 95% CI, 1.92–4.60, *p* < 0.001), and psychological distress (OR = 1.60, 95% CI = 1.25–2.04, *p* < 0.001) in front-line workers, compared to the second-line workers.
Liang (19)	China	Healthcare workers	Depression and anxiety	During	Cross-sectional	Self-reported questionnaire	59	Zung's Self-Rating Anxiety Scale (SAS)Zung's self-rating depression scale (SDS)	Zung's self-rating depression scale showed higher rates of depression in COVID healthcare workers above 30 years old. Zung's self-rating anxiety scale showed no higher rates of anxiety than in other departments.
Xiao et al. (20)	China	Healthcare workers	Anxiety	During	Cross-sectional	Self-reported questionnaire	180	Self-Rating Anxiety Scale (SAS)Pittsburgh Sleep Quality Index (PSQI)	Higher levels of anxiety led to poorer outcomes. Higher levels of social support led to better sleep quality. Lower anxiety led to better outcomes in mental health.
Kang et al. (21)	China	Healthcare workers	Anxiety	During	Cross-sectional	Self-reported questionnaire	994	Patient Health Questionnaire-9 (PHQ-9)General Anxiety Disorder-7 criteria (GAD-7)Insomnia Severity Index (ISI-7)	36.3% had received psychological materials, 50.4% had obtained psychological resources available through media, and 17.5% had participated in group psychological counseling. Those with severe disturbances had accessed fewer psychological materials and psychological resources available through the media. Medical and nursing staff with subthreshold disturbances most wanted to obtain skills to help alleviate others' psychological distress, whereas other medical and nursing staff most wanted to obtain self-help skills. Medical and nursing staff with higher levels of mental health problems were more interested in skills for self-rescue and showed more urgent desires to seek help from psychotherapists and psychiatrists.
Xiao et al. (22)	China	Self-isolated public	Anxiety, sleep	During	Cross-sectional	Self-reported questionnaire	170	Self-Rating Anxiety Scale (SAS), Pittsburgh Sleep Quality Index (PSQI)	Low level of social capital is associated with higher levels of anxiety. Anxiety is associated with stress and lower sleep quality. High level of social capital associated with higher level of sleep quality. With the effect of stress and anxiety, this reduces the effect of social capital on sleep quality.

### Quality Assessment

A total of 36 (37.1%) of the studies are of high quality, 48 (49.5%) are of moderate quality, and 13 (13.4%) are of low quality. The quality assessment results for cohort studies, case–control studies, and cross-sectional studies can be found in Tables 3–5, respectively.

**Table 3:** Newcastle–Ottawa Scale quality assessment for cohort studies (*n* = 18).

**References**	**Selection**				**Comparability**	**Outcome**			**Total score**	**Quality**
	**Representativeness of the exposed cohort**	**Selection of the non-exposed cohort**	**Ascertainment of exposure**	**Demonstration that outcome of interest was not present at start of study**	**Comparability of cohorts on the basis of the design or analysis controlled for confounders**	**Assessment of outcome**	**Was follow-up long enough for outcomes to occur**	**Adequacy of follow-up of cohorts**		**(Low: ≤4; Moderate: 5-6; High: ≥7)**
**General population/Students**										
Cheng (23)			*		**		*	*	5	Moderate
Yu et al. (24)			*	*	**		*		5	Moderate
**Patients/Quarantined**										
Bonanno et al. (25)	*		*	*			*	*	5	Moderate
Chen et al. (26)	*		*	*			*	*	5	Moderate
Cho et al. (27)	*		*	*			*	*	5	Moderate
Hong (28)	*		*	*			*	*	5	Moderate
Hui (29)	*		*	*			*	*	5	Moderate
Lam et al. (30)	*		*	*		*	*	*	6	Moderate
Lee et al. (31)	*	*	*	*	**		*		7	High
Lee (32)	*		*	*			*	*	5	Moderate
Mak et al. (33)	*		*	*		*	*		5	Moderate
Mak et al. (34)	*		*	*		*	*		5	Moderate
Tansey et al. (35)	*		*	*			*	*	5	Moderate
**HCW**										
Chen et al. (36)	*		*	*			*	*	5	Moderate
Lee et al. (37)	*		*	*			*		4	Low
Lung et al. (38)	*		*	*			*	*	5	Moderate
McAlonan et al. (39)	*		*	*			*	*	5	Moderate
Su et al. (40)	*		*	*		*	*	*	6	Moderate

* = 1 star awarded; ** = 2 stars awarded.

**Table 4:** Newcastle–Ottawa Scale quality assessment for case–control studies (*n* = 4).

**References**	**Selection**				**Comparability**	**Exposure**			**Total score**	**Quality**
	**Is the case definition adequate?**	**Representativeness of the cases**	**Selection of controls**	**Definition of controls**	**Comparability of cases and controls on the basis of the design or analysis**	**Ascertainment of exposure**	**Same method of ascertainment for both groups**	**Non-response rate**		**(Low: ≤4; Moderate: 5-6; High: ≥7)**
**General population/Students**										
Lee et al. (41)	*	*		*		*	*	*	5	Moderate
Ng et al. (42)	*	*		*		*	*	*	5	Moderate
**Patients/Quarantined**										
Lee et al. (43)	*	*	*	*	**	*	*		7	High
Han et al. (44)	*	*	*	*		*	*		6	Moderate
**HCW**										
No papers										

* = 1 star awarded; ** = 2 stars awarded.

**Table 5:** Newcastle–Ottawa Scale quality assessment for cross-sectional studies (*n* = 75).

**References**	**Selection**				**Comparability**	**Outcome**		**Total score**	**Quality**
	**Representativeness of the sample**	**Sample size**	**Non-respondents**	**Ascertainment of the exposure (risk factor)**	**The subjects in different outcome groups are comparable, based on the study design or analysis. Confounding factors are controlled**	**Assessment of outcome**	**Statistical test**		**(Low: ≤4; Moderate: 5-6; High: ≥7)**
**General population/Students**									
Al-Rabiaah et al. (45)	*			**		*	*	5	Moderate
Chan et al. (46)	*	*	*	**		**	*	8	High
Cheung et al. (47)	*	*	*	**		**	*	8	High
Cowling et al. (48)	*	*	*	**	**	*	*	9	High
Elizarrarás-Rivas et al. (49)	*		*	**	**	*	*	8	High
Kang et al. (50)	*			**	**	*	*	7	High
Ko et al. (51)	*	*		**		*	*	6	Moderate
Lau et al. (52)	*	*	*	**	**	*	*	9	High
Lee et al. (53)		*		**	**	*	*	7	High
Lee et al. (37)	*	*	*	**		*		6	Moderate
Leung et al. (54)	*			**	**	*	*	7	High
Li et al. (13)	*		*	**		*	*	6	Moderate
Liu et al. (14)			*	**		*	*	5	Moderate
Peng (55)	*		*	**	**	*	*	8	High
Qiu et al. (15)				**	**	*	*	6	Moderate
Quah and Hin-Peng (56)	*		*	**	*	*	*	7	High
Rubin et al. (57)	*			**	**	*	*	7	High
Sim et al. (58)		*		**	**	*		8	High
Sprang and Silman (59)				**	**	*	*	6	Moderate
Wan et al. (60)	*			**		*	*	5	Moderate
Wang et al. (16)				**		*	*	4	Low
Wang et al. (17)			*	**	**	*	*	7	High
Wheaton (61)	*			**	**	*	*	7	High
Wong et al. (62)	*			**		*	*	5	Moderate
Xiao et al. (20)			*	**	**	*	*	7	High
Xu et al. (63)				**	**	*	*	6	Moderate
**Patients/Quarantined**									
Chua et al. (64)		*	*	**	**	*	*	8	High
Cheng^α^ et al. (65)				**		*	*	4	Low
Cheng^β^ et al. (65)	*	*		**		*	*	6	Moderate
Cheng et al. (66)			*	**		*	*	5	Moderate
Hawryluck et al. (7)	*	*		**		*	*	6	Moderate
Jeong et al. (67)			*	**	**	*	*	7	High
Kim (68)	*	*	*	**		**	*	8	High
Kwek et al. (69)	*			**	**	*	*	7	High
Mak WWS et al. (70)				**		*	*	4	Low
Mihashi et al. (71)			*	**		*	*	5	Moderate
Reynolds et al. (72)	*	*	*	**		*	*	7	High
Wang et al. (73)	*	*	*	**	**	*	*	9	High
Wu et al. (8)	*	*		**		*	*	6	Moderate
**HCW**									
Chan and Huak (74)	*	*	*	**		*	*	7	High
Chen et al. (75)				**	**	*	*	6	Moderate
Chen (76)	*	*	*	**		*	*	6	Moderate
Cheng^α^ et al. (65)				**		*	*	4	Low
Chong et al. (77)	*	*	*	**		*	*	7	High
Chua et al. (78)			*	**	**	*	*	7	High
Fiksenbaum et al. (79)				**		*	*	4	Low
Goulia et al. (80)	*	*		**		*	*	6	Moderate
Ho et al. (81)				**		*	*	4	Low
Jung (82)				**		*	*	4	Low
Kang et al. (21)				**	**	*	*	6	Moderate
Khalid (83)	*	*	*	**		*		6	Moderate
Koh (84)	*	*	*	**		*	*	7	High
Lai et al. (18)	*	*	*	**	**	*	*	9	High
Lancee et al. (85)	*	*		**		**	*	7	High
Liang (19)				**		*	*	4	Low
Lin et al. (86)				**		*	*	4	Low
Liu et al. (87)	*			**	**	*	*	7	High
Lu et al. (88)			*	**	**	*	*	7	High
Matsuishi et al. (89)	*	*		**	**	*	*	8	High
Maunder et al. (90)			*	**	**	*	*	7	High
Mishra et al. (91)				**		*	*	4	Low
Nickell et al. (92)				**	**	*	*	6	Moderate
Park et al. (93)		*		**	**	*	*	7	High
Phua et al. (94)				**		*	*	4	Low
Poon et al. (95)	*	*		**		*	*	6	Moderate
Sim et al. (96)				**	**	*	*	6	Moderate
Son (97)	*	*		**		*	*	6	Moderate
Styra et al. (98)	*	*		**		*	*	6	Moderate
Tam et al. (99)				**		*	*	4	Low
Tang et al. (100)				**		*	*	4	Low
Tham et al. (101)				**	**	*	*	6	Moderate
Verma et al. (102)	*	*		**	**	*	*	8	High
Wu et al. (103)		*		**	**	*	*	7	High
Wu et al. (104)				**	**	*	*	6	Moderate
Xiao et al. (22)				**	**	*	*	6	Moderate

α *Adjustment outcomes in Chinese patients following 1 month recovery from severe acute respiratory syndrome in Hong Kong*.

β *Psychological distress and negative appraisals in survivors of severe acute respiratory syndrome (SARS)*.

* = 1 star awarded; ** = 2 stars awarded.

Two studies each looked at two different populations with different sampling methods or study designs and hence were assessed twice under each population category (13, 37). All studies extracted outcomes *via* self-reporting, except for two studies which extracted outcomes *via* records (46, 47).

All of the included studies have a validated tool for assessing mental health outcomes as this was part of our initial exclusion criteria. To assess anxiety, the scales commonly used were Self-Rating Anxiety Scale (SAS) (*n* = 7), State-Trait Anxiety Inventory (STAI) (*n* = 6), Hospital Anxiety and Depression Scale (HADS) (*n* = 6), Generalized Anxiety Disorder-7 (*n* = 4), and Depression Anxiety Stress Scale (DASS) (*n* = 4). To assess psychological distress in general, the scales commonly used were General Health Questionnaire (*n* = 14), 36-Item Short Form Health Survey (*n* = 9), Perceived Stress Scale (PSS) (*n* = 6), and Chinese Health Questionnaire (*n* = 4). To assess PTSD, the commonly used scale was Impact of Event Scale (IES) (*n* = 16). To assess depression, the scales commonly used were Beck Depression Inventory (BDI) (*n* = 6), Center for Epidemiologic Studies Depression Scale (CES-D) (*n* = 6), HADS (*n* = 6), Self-Rating Depression Scale (*n* = 4), and Patient Health Questionnaire-9 (*n* = 4). To assess insomnia, the commonly used scale was Pittsburgh Sleep Quality Index (*n* = 5).

### General Public

A total of 30 papers were identified for the general public.

#### Anxiety Symptoms

In general, public anxiety levels varied with epidemics and countries but were generally low. A high-quality cross-country study reported a significantly lower anxiety level of the public in Singapore compared to Hong Kong (STAI = 1.77 vs. 2.06, *p* < 0.001) during the SARS epidemic (54). In two high-quality studies on H1N1, the reported average general public STAI score in Hong Kong was measured to be 1.8 (48), while only 2.1% of the population in the United Kingdom reported high anxiety (six-item STAI of 18 and more) (57).

Two studies reported decreases in public anxiety associated with more effective dissemination of government information on ongoing epidemics, with a high-quality study demonstrating a significantly lower anxiety in the STAI score of individuals who read government material regarding the epidemic (mean STAI difference = −0.5, 95% CI = −0.9 to −0.05, *p* = 0.03) (16, 57). Three high-quality articles reported that high anxiety was associated with an increased adoption of personal protective measures in three countries: United Kingdom (mean difference 1.7, CI = 1.3 to 2.1, *p* < 0.001), Hong Kong (OR 2.24, CI = 1.27–3.97, *p* < 0.01), and Singapore (OR = 1.140, 95% CI = 1.031–1.283, *p* < 0.05) (54, 56, 57). On the contrary, one high-quality article reported high anxiety being associated with a decreased adoption of such measures instead, suggesting that lower compliance to measures leads to high anxiety (48). However, it is worth mentioning that, in this study, the group with the highest anxiety is more likely to clean and disinfect the house (OR = 1.41, 95% CI = 1.13–1.76).

The risk factors of higher anxiety identified from high/moderate-quality studies include female gender (48), low social capital (22), contact with suspected cases (63), and staying in close proximity to hospitals (50, 62). Interestingly, one moderate-quality case–control study particularly looked at pregnant women before and during SARS, identifying elevated anxiety levels in mothers during the SARS outbreak (mean STAI = 37.2 vs. 35.5, *p* = 0.02) (41). Moreover, 92% of the women surveyed refrained from leaving the house, and 70% worried about the possible teratogenicity of treatment should it be required.

#### PTSD Symptoms

Several risk factors for PTSD were identified from high/moderate-quality studies: children with parents having PTSD (59), low education level (52), female gender (14, 63), older age (53), and proximity to outbreak-prevalent regions (53, 63). One moderate-quality study on COVID-19 reported better sleep quality in those with lower PTSD Checklist for DSM-5 (PCL-5) scores (*p* < 0.05) (14). Interestingly, one moderate-quality COVID-19 study identified higher Vicarious Trauma Scale scores in the general public when compared to front-line nurses [75.5 (95% CI = 62–88.3) vs. 64 (95% CI = 52–75), *p* < 0.001] (13).

#### Depression Symptoms

In terms of depression, one moderate-quality cohort study reported an increase in CES-D score during SARS as compared to the participants' baseline before SARS (mean CES-D = 12.94 vs. 10.74, *p* < 0.05) (24), while one moderate-quality case–control study of pregnant women showed no significant difference in BDI score between the pre-SARS and post-SARS cohort (7.8 vs. 8.7, *p* = 0.16) (41). A study in Taiwan demonstrated that the Taiwanese Depression Questionnaire score was significantly higher if the family or friends were affected (quarantined or contracted) by SARS (*t* = 7.95, *p* < 0.001) (51). The risk factors of depression from the moderate-quality studies include age ≥60 years (53), personal perception of risk of infection (14), financial loss (14, 52), and directly impacted by SARS (52).

#### Population Subgroups

All studies covering the subgroup population are of moderate/low quality. One subgroup identified to have the highest risk by a moderate-quality study is migrant workers (mean = 31.89, *F* = 1,602.501, *p* < 0.001) due to the financial impact and perceived risk of infection from long-distance travels (15). Another subgroup identified by a low-quality study is university students, where they scored significantly higher for IES (*B* = 0.20, 95% CI = 0.05–0.35), DASS stress subscale (*B* = 0.11, 95% CI = 0.02–0.19), and DASS anxiety subscale (*B* = 0.16, 95% CI = 0.02–0.30) when compared to the employed population (16). Among five studies for undergraduate students, two moderate-quality studies are specific to healthcare students (45, 62). One study compared medical students to non-medical faculties in the same school and non-medical faculties in another school. It was found that the medical students have significantly higher mean SAS scores (34.05 vs. 33.43 vs. 31.71, *p* < 0.01) (62).

The last subgroup at risk is the chronically ill patients. Although one moderate-quality study showed high levels of anxiety and depression in thoracic surgery patients on waitlist during SARS, it was unable to demonstrate statistical significance (*p* = 0.582 for anxiety, *p* = 0.841 for depression) (60). When psychological support is provided, one moderate-quality study reported lower depression rates of 5.5% compared to a meta-analysis data of 20%, while another moderate-quality study reported a significantly lower Brief Symptom Inventory score in depression subsection only [*F*_(1, 28)_ = 5.215, *p* < 0.05] (37, 42). However, these two studies had a small sample size.

#### Risk of Older Individuals

Older individuals have a higher risk of developing a psychological disease (15, 46, 47, 49, 52, 53). In healthy individuals, increasing perceived stress levels is associated with increasing age (Spearman's rho 0.33, *p* < 0.005, Bonferroni-corrected) (64). One high-quality study identified older age as an association for high levels of depression (0.05 CI = 0.04–0.07, *p* < 0.001) and death anxiety (0.32 CI = 0.23–0.41, *p* < 0.001) during H1N1 when their family members were in the intensive care unit (49). The possibility of losing a younger family member may explain the high rates. On the extreme end of the mental health spectrum, two high-quality papers by the same author identified the significantly higher suicide rates among Hong Kong's elderly, ≥65 years of age, during the period coinciding with the SARS outbreak in April 2003 (April 2001 IRR = 0.362, *p* = 0.002; April 2002 IRR = 0.548, *p* = 0.032) (46, 47). The higher level of suicide was found to persist for a year after SARS (2004 IRR = 0.835, *p* = 0.045).

### Healthcare Workers

A total of 41 papers were identified for healthcare workers.

#### Anxiety Symptoms

During epidemics, the development of anxiety symptoms is chiefly propelled by the healthcare workers' consistently high exposure to infected patients. One paper reported healthcare workers to have higher STAI scores compared to administrative staff (mean = 51.1 vs. 47.1, *p* < 0.001) and higher STAI scores among healthcare workers exposed to patients with SARS than those not exposed (mean = 52.6 vs. 49.8, *p* < 0.001). The same paper reported that a greater proportion of exposed compared to non-exposed healthcare workers had discomfort from wearing a protective gear (4.1 vs. 2.9%, *p* < 0.001), worry of being infected (2.0 vs. 1.8%, *p* < 0.001), worry of infecting others (2.0 vs. 1.7%, *p* < 0.001), and perceived prejudice from others (1.2 vs. 0.9%, *p* < 0.001) (95). All these factors could explain why healthcare workers with a high exposure to infected patients are at a higher risk of anxiety symptoms.

Fear of transmitting the virus to family members was consistently reported as a leading cause of anxiety (45, 80, 92, 105). Two of the studies identified the rate of fear to be around 60% of the respondents (80, 92). Another cited study reported that females are more likely to be worried about family transmission compared to males, with higher reported anxiety scores (mean = 3.67 vs. 2.16, *p* < 0.05) (105). Among non-physicians, this fear was compounded by the perceived threat of mortality imposed by the respiratory virus itself according to a Canadian study of 333 nurses as measured *via* the emotional exhaustion subscale of the Maslach Burnout Inventory (79).

Anxiety among healthcare workers was propelled by traits of neuroticism. In one high-quality and one low-quality study, if workers lacked maternal care or were overprotected by their mothers, they would have poorer mental health outcomes after the epidemic (38, 88). A high-quality Taiwanese study demonstrated significant neuroticism among a sample group of 24 physicians as measured on the Eysenck Personality Questionnaire (mean = 2.75), who also scored high on the Chinese Health Questionnaire (mean = 1.63) across three domains of anxiety, somatic symptoms, and depression (88). This is supported by another study of moderate quality where neuroticism is associated with worse mental health outcome on the same scale (β = 0.44, SE = 0.06, *p* < 0.001) (38).

#### PTSD Symptoms

Fear of transmission of respiratory viruses to family members, especially their children, is a significant factor for the development of PTSD, though both studies reported that this was of low quality (81, 106). A Hong Kong study showed that, using the SARS Fear Scale (SFS) score, the fear item of worry about family being infected had the highest mean score (2.24 ± 0.56, *p* = 0.483 on a four-point Likert scale) in a sampled group of 82 healthcare workers (81). The correlation analysis showed that the three subscales of SFS scores were positively correlated with the three subscales of the Chinese version of IES-R (*p* < 0.01), and the total scores of scales had *r* = 0.64 and *p* < 0.01.

Consistent contact with patients was another major risk factor in two high-quality, one moderate-quality, and one low-quality study (65, 80, 93, 102). Elevated rates of PTSD were reported in all healthcare professions, as supported by one high-quality and one low-quality study (37, 107), especially those who work in high-risk areas (77, 89, 98) such as the Emergency Department (86) and respiratory medicine department (39) or those who were quarantined (72). One high-quality study attributed it to the workers' exhaustion, lethargy, and high workload (89). The nurses in this aforementioned Japanese study, who felt more exhaustion (*B* = 0.34, SE = 0.12, β = 0.14, *p* = 0.004) and workload (*B* = 0.34, SE = 0.07, β = 0.21, *p* < 0.001) than doctors, also had higher total IES scores than that of doctors (nurses: *B* = 0.90, SE = 0.32, β = 0.14, *p* = 0.005) (89). High-risk workers with PTSD symptoms retrospectively reported fatigue (70.3%, compared with 22.1% of low-risk workers; χ^2^ = 37.9, *p* < 0.05), poor sleep (30.2%, compared with 7.4% of low-risk workers; χ^2^ = 12.7, *p* < 0.05), health anxiety (57.3%, compared with 41.2%; χ^2^ = 4.1 of low-risk workers, *p* < 0.05), and fear of social contact (41.7%, compared with 23.5% of low-risk workers; χ^2^ = 5.8, *p* < 0.05) in a moderate-quality study (39).

In one high-quality and one low-quality study, non-modifiable risk factors of young age and inexperience were highlighted as contributors to PTSD (77, 100). One study reported higher PTSD Checklist-Civilian Version scores in healthcare workers aged 20–30 years compared to those aged above 40 years (mean = 1.87 vs. 1.51, *p* < 0.05) (100). Furthermore, access to beneficial psychological material had shown to reduce PTSD symptoms. A moderate-quality study of the COVID-19 pandemic in Wuhan reported that 17.7% in a sampled group who accessed psychological material had a mean IES-R score of 6.1 (*p* < 0.001) vs. 41.4% in another sampled group who accessed psychological material who had a mean IES-R score of 60.0 (*p* < 0.001) (21). Other predictors of acquiring PTSD from high-quality studies include maladaptive coping strategies (90, 94), attachment anxiety (90), and singlehood (74).

#### Depression Symptoms

A previous positive history for psychiatric disorders was predictive of developing a mood disorder during an epidemic by one high-quality (χ^2^ = 8.0, df = 1, 1, *p* < 0.01) and one moderate-quality study (β = 0.22, *p* = 0.02) (40, 85). Aside from the aforementioned risk factors for PTSD which have a component of depression, post-epidemic depression was closely linked to workers having traumatic experiences pre-outbreak as highlighted in a high-quality study (87). In this study, a multinomial logistic regression model of having had pre-SARS traumatic experiences revealed an adjusted odds ratio of 3.39 in the high depressive symptom group compared to the low depressive symptom group (CI 1.47–7.84, *p* = 0.004) (87).

#### Comparing Nurses and Physicians

Nurses showed a higher prevalence for psychiatric symptoms when compared to physicians in a high-quality (depression 7.1 vs. 4.9%, *p* = 0.01) (18) and a moderate-quality (psychological distress OR = 2.2, 95% CI = 0.59–2.07, *p* = 0.046) study, respectively (80). However, two other moderate-quality studies showed that nurses may have had better mental health outcomes due to better working environments and being adequately trained (38, 88). In Taiwan, it is postulated that anxiety in physicians was compounded by local medical disputes and criminal law (38, 88). This is corroborated by a Chinese study which showed higher rates of somatization in physicians than in nurses (β = −0.15, *p* = 0.034) (38).

Among nurses and doctors, it is worth noting that a recent high-quality COVID-19 study reported significantly higher symptoms of depression (OR = 1.52, 95% CI = 1.112.09, *p* = 0.01), anxiety (OR = 1.57, 95% CI = 1.22–2.02, *p* < 0.001), insomnia (OR = 2.97, 95% CI = 1.92–4.60, *p* < 0.001), and psychological distress (OR = 1.60, 95% CI = 1.25–2.04, *p* < 0.001) in front-line workers compared to second-line workers (18).

#### Isolation and Stigmatization

The listed causes for workers being in social isolation include being quarantined (104), isolation from family members (77, 80), and voluntary restriction from social contacts (80). In a moderate-quality Taiwanese study, self-isolation caused fatigue, loneliness, frustration, and anxiety, which contributed to higher psychological morbidity (75). From the study in which a survey was distributed after 4 weeks of quarantine with SARS patients, the duration of time in contact with infected patients was closely associated with the negative affectation in mental and emotional health of healthcare workers in major subscales and predicted their mental health outcomes (adjusted *R*^2^ = 0.069; *p* = 0.038) (75). They fared worse across domains of emotional role, mental health, and social functioning. These domains were closely associated with increased contact days, contact hours, and contact hours-per-day with SARS patients (75).

Stigmatization of healthcare workers through restriction of social contacts led to increased anxiety symptoms in one high-quality and two moderate-quality studies (45, 80, 93). This stigmatization had therefore resulted in healthcare workers being treated differently (92) and has led to subsequent rejection by their neighborhoods. In the high-quality study, receiving different treatments from the public by virtue of being a healthcare worker was closely associated with higher levels of anxiety symptoms of concern for personal and family health (adjusted OR 1.6, 95% CI = 1.2–2.1) according to a logistic regression analysis (92).

#### Long-Term Impact

In five studies, psychological morbidity remained prominent post-epidemic in a small proportion of healthcare workers shown by two high-quality and two moderate-quality studies (38, 85, 103, 104). A high-quality study reported a new onset or worsening of panic disorder discovered in a handful of SARS physicians in Canada 13–22 months post-epidemic (85). In Taiwan, while most workers reported no significant sources of daily life stress 3 years after SARS, 15.4% of the sampled workers still displayed psychological symptoms (χ^2^ = 2.14, *p* = 0.343). Though statistically significant, a multiple linear regression result by the same study showed that this was associated with daily-life stressors (β = 1.07, SE = 0.31, *p* = 0.001) rather than the SARS crisis (38). In Beijing, 10% of the sampled workers had high PTSD symptoms (IES-R ≥ 20) after 3 years in one high-quality and one moderate-quality study by the same author (103, 104). The latter study attributed this to quarantine during the SARS period (OR = 3.47, 95% CI = 1.93–6.25, *p* < 0.0001), friends or family being affected by SARS (OR = 3.74, 95% CI = 1.83–7.62, *p* = 0.0003), or close contact with SARS patients (OR = 3.11, 95% CI = 1.76–5.49, *p* < 0.0001). Among the individuals with high PTSD, the latter study also identified a higher risk of alcohol dependence in those individuals with high PTSD symptoms in a regression analysis (OR = 1.65, 95% CI = 1.02–2.66) (103).

### Patients of the Viral Respiratory Illness

There were 20 studies identified for patients.

#### Long Term and Short Term

Compared to non-patients, patients of epidemics had worse mental health outcomes in both the short term (26, 31, 65, 108) and the long term (25, 26). It was reported that the PSS scores were significantly higher in patients during the epidemic (mean = 19.8 vs. 17.9, *p* < 0.01) and 1 year after the outbreak (mean = 19.9 vs. 17.3, *p* < 0.01) (31). Even after the epidemics, two moderate-quality studies reported the persistence of psychological distress in survivors at 18 months (25) and 24 months (26) after the outbreak.

#### Associated Factors

Factors positively associated with symptoms of psychological distress, anxiety, depression, and PTSD extracted from high-quality and moderate-quality studies include female gender (8, 25, 31, 33, 66, 108), patients who were healthcare workers (31, 33, 34, 65, 66, 108), having poor social support (8, 33, 70), perception of being stigmatized during the outbreak (30, 33), knowing someone who had SARS (109), and losing a family member to SARS (108). It was reported that, during the epidemic, females scored higher in PSS (mean = 20.7 vs. 18.0, *p* < 0.05), DASS (depression mean = 13.1 vs. 7.8, *p* < 0.01; anxiety mean = 12.5 vs. 7.0, *p* = 0.001), and IES-R (intrusion mean = 1.6 vs. 1.1, *p* < 0.01; avoidance mean = 1.3 vs. 0.9, *p* < 0.05; hyperarousal mean = 1.4 vs. 0.9, *p* < 0.05) (31). Compared to non-healthcare workers, healthcare workers were reported to have higher scores in DASS (depression mean = 15.1 vs. 9.0, df = 3, 86, *F* = 3.9, *p* < 0.01; anxiety mean = 14.6 vs. 8.2, df = 3, 85, *F* = 5.2, *p* = 0.001) and IES-R (intrusion mean = 2.0 vs. 1.1, df = 3, 85, *F* = 5.7, *p* < 0.001; avoidance mean = 1.5 vs. 0.9, df = 3, 85, *F* = 3.5, *p* < 0.05; hyperarousal mean = 1.7 vs. 1.0, df = 3, 85, *F* = 3.5, *p* < 0.05) (31). In terms of healthcare workers, it was postulated that this was because the healthcare workers' workplace was also where they had such bad experiences as a patient. In addition, healthcare workers may have a lowered self-esteem as they perceive themselves to be “virus spreaders” (108).

Factors negatively associated with symptoms of psychological distress, anxiety, depression, and PTSD extracted from moderate-quality and low-quality studies include increased duration after the end of the epidemic (34, 35) and increased education levels of the patient (70). One study reported that, over a period of 30 months, 23 of 53 subjects (43.4%) recovered from DSM-IV psychiatric disorders diagnosed post-SARS (34).

#### Miscellaneous Outcomes

Several interesting outcomes reported are worth mentioning. A high-quality case–control study of SARS patients with psychosis reported that a family history of psychiatric illness was associated with an increased incidence of SARS-related psychosis in the short term (33 vs. 0%, *p* = 0.02) (43). One moderate-quality study identified chronic fatigue syndrome which persisted at the fourth year of follow-up. Active psychiatric illness was found to be significantly associated with patients with chronic fatigue syndrome. One study reported that 39 of 51 patients (76.5%) with active psychiatric illness had chronic fatigue syndrome (30). Interestingly, one moderate-quality study of patients reported a higher incidence of narcolepsy during and shortly after the influenza A/H1N1 pandemic, independent of H1N1 vaccinations. It was reported that the incidence of narcolepsy following the 2010 pandemic was 3.2 times greater than forecasted (*p* < 0.001) (44).

#### Significant Comorbidities or Complications

Six studies of varying qualities on SARS and influenza A (H7N9) patients reported that patients with significant comorbidities or complications had higher levels of psychological distress (25, 26, 65, 69), depression symptoms (108), and PTSD symptoms (33).

In the short term, a high-quality, a moderate-quality, and a low-quality study, respectively, reported that pre-existing chronic disease, perceived severity of SARS symptoms, use of steroids for respiratory complications, and ICU admission were associated with higher levels of psychological distress (65, 69) and depression symptoms (108). In the long term, three moderate-quality studies reported that a pre-existing chronic disease, poorer perceived physical health, higher average pain, patients who had acute respiratory distress syndrome, and patients who had avascular necrosis as a complication of steroid treatment were associated with higher levels of psychological distress (25, 26) and PTSD symptoms (33). It was reported that chronic medical illnesses (OR = 7.44, 95% CI = 1.44–38.59, *p* = 0.014) and avascular necrosis (OR = 4.53, 95% CI = 1.41–14.50, *p* = 0.010) were predictors of PTSD (33). ICU admission and having avascular necrosis were postulated to cause psychological distress by resulting in activity restriction and functional impairment in one high-quality and one moderate-quality study, respectively (33, 69).

Interestingly, steroid treatment was associated with short-term psychological distress in a low-quality study (65) and psychosis in a high-quality study (43). The median cumulative dose of hydrocortisone was significantly higher in patients with SARS-related psychosis than in non-psychotic subjects (10,975 vs. 6,780 mg, *p* = 0.017) (43). This is in keeping with the findings that high-dose steroids can cause mood fluctuation and cognitive distortion, even in the absence of physical complications (64, 110).

### Quarantined Individuals

Six studies were identified for quarantined individuals. Three high-quality and one moderate-quality study, respectively, reported mental health outcomes during quarantine (7, 67, 72, 73), and two moderate-quality studies reported mental health outcomes after quarantine (27, 71).

#### Comparison to Non-quarantined Individuals

Only two papers compared the levels of psychological distress and PTSD symptoms among quarantined vs. non-quarantined individuals (71, 73). Both papers, one of high-quality and one of moderate-quality, reported that the mental health outcomes were not significantly different between both groups. Interestingly, quarantined females had lower levels of PTSD symptoms during the epidemic as compared to non-quarantined females in the high-quality study (OR = 0.24, 95% CI = 0.07–0.83, *p* < 0.05) (73).

In two moderate-quality studies, the quarantined individuals, while not shown to be at a higher risk of PTSD as compared to non-quarantined individuals, described a sense of isolation due to the lack of physical contact with family members, activity restriction, and not being able to shop for basic necessities (7, 71). The infection control measures imposed caused physical discomfort, feelings of isolation, and anxiety (7). These factors could have contributed to certain groups of quarantined individuals having poorer mental health outcomes compared to non-quarantined individuals.

#### During Quarantine

Factors positively associated with symptoms of anxiety in a high-quality paper include having a personal history of psychiatric disorders (RR = 5.3, 95% CI = 2.511.0) and financial loss (RR = 1.9, 95% CI = 1.4–2.6) (111). A longer duration of quarantine is also shown to have worse mental health outcomes in a high-quality and a moderate-quality study, respectively (7, 66). It was reported that IES-R was correlated with a longer duration of quarantine (β = 0.40, *p* = 0.012) (72).

#### After Quarantine

In one moderate-quality study, being female was positively associated with symptoms of PTSD compared to males (IES-Revised-Korean Version sleep disturbance mean = 1.57 vs. 0.46, *p* = 0.024) (27). In another moderate-quality study, the factors positively associated with psychological distress were cessation of work and income reduction (OR = 9.9, 95% CI = 4.4–21.9, *p* = 0.000) (71) and experiencing symptoms related to the epidemics (OR = 7.9, 95% CI = 1.5–41.9, *p* = 0.016) (71). Interestingly, a shorter duration of quarantine was associated with higher levels of PTSD symptoms after quarantine (27), which is the opposite of what was reported during quarantine.

## Discussion

### Internal and External Validity

The strength of our study lies in our broad search and stringent selection criteria for our papers. In our search, we included all controlled vocabulary and keywords of diagnoses to capture a comprehensive list of psychiatric outcomes. We excluded papers reporting an outbreak before year 2000, which may compromise external validity, papers with potential confounders like vaccination during an epidemic, and papers with unvalidated scales, which may report potentially subjective and inaccurate results.

Most of the studies involving the general public took place during or immediately after the epidemic and are cross-sectional in nature. As such, the long-term psychiatric morbidities in the general public were not well-studied compared to healthcare workers and patients. Cohort studies should be conducted to follow up with these populations to establish if mental health disturbances still persisted after the epidemics.

In general, we note that the scales used are an effective screening tool for mental health conditions but are largely not diagnostic. Different studies adopted different tools for the same mental health outcome. Even among studies using the same scale, different cutoff points for the same disease were reported. This could have accounted for the variability in prevalence of high-risk individuals identified.

Due to the cross-sectional nature of our study, the cause-and-effect relationship between risk factors and mental health outcome is frequently poorly established. The samples in these studies do not have a control population as everyone in the region or nation would have been through the epidemic (112). Furthermore, many studies have been subjected to recall bias. This is considering that, in some studies conducted, there was a substantial time lapse between the epidemic and the time at which individuals reported their psychology during the epidemic. Because an overwhelming majority of the studies considered were voluntary, non-respondent bias could have set in if individuals who responded to the surveys had a significant but unreported difference in psychology compared to those who declined or did not respond to the surveys. In terms of data collection, many studies used written or online questionnaires. Selection bias is present as illiterate and less tech-savvy individuals are unable to complete the questionnaires. Moreover, the severely ill are less likely to participate in the questionnaires. Sampling bias is present as some studies reported using non-randomized sampling methods such as snowball sampling and convenience sampling. Because of social desirability bias, the participants may under-report symptoms or behaviors they deem less socially acceptable during an epidemic.

Multiple studies have examined the effects of respiratory illnesses [e.g., legionnaires' disease (113), community-acquired pneumonia (114), and acute respiratory distress syndrome (115)] on the mental health of patients in non-epidemic settings. Severe psychiatric morbidities were shown in survivors, including PTSD, anxiety, depression, and chronic fatigue syndrome, and in one study, PTSD still persisted at the 8-year follow-up (115). Noting how similar the psychological course of these patients is to patients in respiratory epidemics, it may be worth to investigate a common broad approach toward mental health intervention for all patients who have been through a severe respiratory disease. As a new recommendation, this approach should emphasize heavily on the anticipation and the management of PTSD after the patients had recovered. For example, a comprehensive screening and referral policy by the psychiatry department could be introduced for all patients recovering from severe respiratory illnesses.

### Heterogeneity

Many articles during the epidemic variably assessed their participants during its beginning, peak, or tail-end, which can lead to unaccounted differences in mental health responses.

Different levels of prevalence of high-risk scores for PTSD have been reported, with lowest being 7% in COVID-19 (14) and H1N1 (59) and highest being 26% in SARS (58). This could be accounted for by various factors such as transmissibility and varying case-fatality rates between different respiratory viruses (116). The differences in containment efforts and method of information dissemination between countries may account for the variability as well.

### Demographics

Females were at a higher risk for the many mental health outcomes aforementioned. In the same vein, a recent study reported that being male is a protective factor for depression and anxiety (117). In terms of social factors, traditional gender roles could be upheld in many countries surveyed which had a conservative, even patriarchal, background (118). Strong child-centric ideals in many of such countries could have meant that mothers had to pay markedly more attention to the well-being of their children and families before themselves. Specifically for PTSD, the higher risk may be attributed to the differences in fear mechanisms (119) between sexes and the higher genetic heritability (111) in females.

Among healthcare workers, youth and inexperience were associated with poorer mental health outcomes (85, 99, 100). We postulate that they face pressure adapting to a new healthcare system and new stressors from an epidemic. One study showed higher resilience in older healthcare workers because of better work–life balance and higher personal accomplishment, possibly leading to better mental health (120).

Conversely, among the general public and the quarantined, old age was associated with worse outcomes. This was postulated to be because older subjects were cognizant of poorer prognosis if infected (64). Higher perception of risk causes them to adopt more protective measures and leads to anxiety (121, 122), which correspond to hyperarousal and avoidant behaviors of PTSD symptoms.

### Recommendations for the COVID-19 Pandemic

In view of the aggressive lockdown strategies employed by countries, officials should consider the mental health problems (123) weighing against its epidemiological benefits. In our systematic review, the only two studies that looked at the mental health outcomes between quarantined and non-quarantined individuals reported no significant difference between the groups. Unlike in quarantine, lockdowns have devastating economic impact and are subjected to unexpected extensions.

Recession is a major cause of depression during epidemics as aforementioned (24, 51). COVID-19-related suicides are on the rise (124), with one Pakistan study attributing this to the lockdown-related economic instability and high unemployment rates (5). Financial assistance should also be provided to individuals affected by the market downturn. Social support funds may ease psychological distress and burden for families or businesses.

Drawing on past trends and recent studies, more attention should be given to the vulnerable groups identified at risk of poor mental health outcomes during epidemics, including older individuals, migrant workers, students, and chronically ill patients. We had seen higher suicide rates in the elderly during the SARS epidemic (46, 47), with affective disorders being a significant risk factor in this age group (125). To prevent this, telephone-based or online trauma-focused psychotherapy can be deployed, with strong outreach efforts, to these vulnerable groups.

As shown in our results and discussed above, females are at a higher risk for a psychological disease. Among a small group of females, the increased prevalence of mental health disorders during the COVID-19 pandemic may be accounted for by increased domestic violence because of home isolation (126). Increased surveillance of domestic violence could be enacted *via* frequent checks *via* telemedicine consultations and *via* an increased index of suspicion for females presenting with non-accidental injuries to primary care.

Among the general public, anxiety can be eased with the officials providing accurate and timely information as seen in identified studies (15, 16, 57). To reduce further distress, the relevant authorities should dispel rumors that could spread fear (127). Proper channels for communication *via* appropriate media should be updated with the latest and most accurate information.

Stigmatization of some members of the public and healthcare workers is a pertinent and recurring issue. These individuals, including those of Asian descent in COVID-19 epidemic (128, 129), are allegedly labeled or shunned because they are perceived as culpable of transmitting the virus. This prejudice simply because of race or profession confers a significant psychological burden onto affected individuals. Ending the stigmatization should begin with denouncement of such behavior by governments. In a period of high stress and uncertainty in this rapidly evolving global health crisis, compassion, and empathy, instead of dissent and distrust, will bring better mental health outcomes to the world.

We note that healthcare workers suffering from psychological disorders had a largely positive prognosis, even up to 6 months post-epidemic (101). The resilience of these workers' mental health in past epidemics was buoyed by a positive work culture with mental health support and crisis preparation (20, 36, 74, 78, 94, 101). This encouraging finding highlights the importance of fostering a culture at work that nurtures the mental health of healthcare workers. Proper avenues for workers to seek psychological help to develop better coping strategies should be made available (117, 130). With their psychological needs taken care of, healthcare workers can continue to serve patients compassionately (21). Unity and social support among healthcare workers in the face of crises (20, 78, 96) play a crucial role in helping workers cope effectively. A multimodal approach to crisis preparation using seminars, practical workshops, and simulation exercises could reduce anxiety in physicians should a future epidemic be imminent (91).

## Conclusion

This study has shown that the general public, healthcare workers, patients, and quarantined individuals in many countries suffer from many stresses during respiratory epidemics that have poor implications on mental health, even long after the epidemic. These psychological symptoms, if not detected and managed early, can progress into full-blown psychiatric conditions. In applying this knowledge to the COVID-19 epidemic, it would be prudent for governments to step up and use resources to implement policies specifically designed for each high-risk group. These policies will serve to relieve the psychological burden and provide better well-being for all.

## Data Availability Statement

All datasets generated for this study are included in the article/Supplementary Material.

## Author Contributions

YL, CRC, ZX, RH, and CH contributed to the study design, writing, and approval of the manuscript. YL, CRC, and ZX contributed to the literature search, data extraction, and analysis. All authors contributed to the article and approved the submitted version.

## Conflict of Interest

The authors declare that the research was conducted in the absence of any commercial or financial relationships that could be construed as a potential conflict of interest.
