# Supplementary material for: A Systematic Review of the Impact of Viral Respiratory Epidemics on Mental Health: An Implication on the Coronavirus Disease 2019 Pandemic
Source: Front Psychiatry. 2020 Nov 23;11:565098. doi: 10.3389/fpsyt.2020.565098 (PMC7719673; doi:10.3389/fpsyt.2020.565098)
Supplement: Supplementary file 1 [file Data_Sheet_1.DOCX]

**Appendix A Search strategy and results**

A search was conducted on 4th April 2020 on Pubmed (2333), Embase (3011), PsycINFO (440), CINAHL (722), Scopus (3317), Web of Science (4938), and Cochrane (506).

**Table A.1 Pubmed**

| No. | Searches | Results |
| --- | --- | --- |
| #1 | (COVID-19[MeSH Terms] OR Severe acute respiratory syndrome coronavirus 2[MeSH Terms] OR Middle East Respiratory Syndrome Coronavirus[MeSH Terms] OR Severe Acute Respiratory Syndrome[MeSH Terms] OR SARS Virus[MeSH Terms]) OR (COVID-19[Title/Abstract] OR COVID19[Title/Abstract] OR 2019 novel coronavirus infection[Title/Abstract] OR 2019-nCoV infection[Title/Abstract] OR COVID-19 pandemic[Title/Abstract] OR coronavirus disease-19[Title/Abstract] OR 2019-nCoV disease[Title/Abstract] OR 2019 novel coronavirus disease[Title/Abstract] OR coronavirus disease 2019[Title/Abstract] OR severe acute respiratory syndrome coronavirus 2[Title/Abstract] OR Wuhan coronavirus[Title/Abstract] OR Wuhan seafood market pneumonia virus[Title/Abstract] OR COVID19 virus[Title/Abstract] OR COVID-19 virus[Title/Abstract] OR coronavirus disease 2019 virus[Title/Abstract] OR SARS-CoV-2[Title/Abstract] OR SARS2[Title/Abstract] OR 2019-nCoV[Title/Abstract] OR 2019 novel coronavirus[Title/Abstract])) OR (MERS[Title/Abstract] OR MERS-CoV[Title/Abstract] OR MERS Virus[Title/Abstract] OR MERS Viruses[Title/Abstract] OR Virus, MERS[Title/Abstract] OR Viruses, MERS[Title/Abstract] OR Middle East respiratory syndrome-related coronavirus[Title/Abstract] OR Middle East respiratory syndrome related coronavirus[Title/Abstract])) OR (SARS[Title/Abstract] OR Severe Acute Respiratory Syndrome Virus[Title/Abstract] OR SARS-Related Coronavirus[Title/Abstract] OR Coronavirus, SARS-Related[Title/Abstract] OR SARS Related Coronavirus[Title/Abstract] OR SARS-CoV[Title/Abstract] OR Urbani SARS-Associated Coronavirus[Title/Abstract] OR Coronavirus, Urbani SARS-Associated[Title/Abstract] OR SARS-Associated Coronavirus, Urbani[Title/Abstract] OR Urbani SARS Associated Coronavirus[Title/Abstract] OR SARS Coronavirus[Title/Abstract] OR Coronavirus, SARS[Title/Abstract] OR Severe acute respiratory syndrome-related coronavirus[Title/Abstract] OR Severe acute respiratory syndrome related coronavirus[Title/Abstract] OR SARS-Associated Coronavirus[Title/Abstract] OR Coronavirus, SARS-Associated[Title/Abstract] OR SARS Associated Coronavirus[Title/Abstract]) | 15664 |
| #2 | (Influenza, Human[MeSH Terms]) AND Disease Outbreaks[MeSH Terms] | 12694 |
| #3 | ((Influenza[Title/Abstract] OR Influenzas[Title/Abstract] OR Human Influenzas[Title/Abstract] OR Influenzas, Human[Title/Abstract] OR Human Flu[Title/Abstract] OR Flu, Human[Title/Abstract] OR Human Influenza[Title/Abstract] OR Influenza in Humans[Title/Abstract] OR Influenza in Human[Title/Abstract] OR Grippe[Title/Abstract])) AND (Disease Outbreaks[Title/Abstract] OR Disease Outbreak[Title/Abstract] OR Outbreak, Disease[Title/Abstract] OR Outbreaks, Disease[Title/Abstract] OR Outbreaks[Title/Abstract] OR Infectious Disease Outbreaks[Title/Abstract] OR Disease Outbreak, Infectious[Title/Abstract] OR Disease Outbreaks, Infectious[Title/Abstract] OR Infectious Disease Outbreak[Title/Abstract] OR Outbreak, Infectious Disease[Title/Abstract] OR Outbreaks, Infectious Disease[Title/Abstract] OR Pandemic[Title/Abstract] OR Pandemics[Title/Abstract] OR Epidemic[Title/Abstract] OR Epidemics[Title/Abstract]) | 27015 |
| #4 | #1 OR #2 OR #3 | 45994 |
| #5 | ((Mental health[MeSH Terms]) OR Mental disorders[MeSH Terms]) OR (Mental Health[Title/Abstract] OR Mental Hygiene[Title/Abstract] OR Psychological Health[Title/Abstract] OR Psychological Hygiene[Title/Abstract] OR Mental Disorder[Title/Abstract] OR Psychiatric Disorder[Title/Abstract] OR Psychiatr*[Title/Abstract] OR Psychosis[Title/Abstract] OR Schizophreni*[Title/Abstract] OR Delusion*[Title/Abstract] OR Paranoi*[Title/Abstract] OR Mania[Title/Abstract] OR Manic[Title/Abstract] OR Hypomania[Title/Abstract] OR Bipolar Disorder[Title/Abstract] OR Bipolar[Title/Abstract] OR Mood Disorder[Title/Abstract] OR Mood Disorders[Title/Abstract] OR Depress*[Title/Abstract] OR Depressive Disorder[Title/Abstract] OR Major Depressive Disorder[Title/Abstract] OR Cyclothymia[Title/Abstract] OR Dysthymia[Title/Abstract] OR Phobi*[Title/Abstract] OR Fear[Title/Abstract] OR Agoraphobi*[Title/Abstract] OR Generalised Anxiety Disorder[Title/Abstract] OR Generalized Anxiety Disorder[Title/Abstract] OR Anxiety Disorder[Title/Abstract] OR Anxiet*[Title/Abstract] OR Panic Disorder[Title/Abstract] OR Panic Attack[Title/Abstract] OR Panic[Title/Abstract] OR Obsessi*[Title/Abstract] OR Compulsi*[Title/Abstract] OR Obsessive Compulsive Disorder[Title/Abstract] OR OCD[Title/Abstract] OR Stress[Title/Abstract] OR Post Traumatic Stress Disorder[Title/Abstract] OR PTSD[Title/Abstract] OR Adjustment Disorder[Title/Abstract] OR Adjustment[Title/Abstract] OR Dissociative Disorder[Title/Abstract] OR Dissociat*[Title/Abstract] OR Somatoform Disorder[Title/Abstract] OR Somatoform[Title/Abstract] OR Hypochondria*[Title/Abstract] OR Depersonali*[Title/Abstract] OR Dereali*[Title/Abstract] OR Sleep Disorder[Title/Abstract] OR Sleep[Title/Abstract] OR Insomnia[Title/Abstract] OR Hypersomnia[Title/Abstract] OR Sleep Terror[Title/Abstract] OR Nightmare[Title/Abstract] OR Sleepwalking[Title/Abstract] OR Sexual Dysfunction[Title/Abstract] OR Sexual[Title/Abstract] OR Motor Function[Title/Abstract] OR Developmental[Title/Abstract] OR Autism[Title/Abstract] OR Autism Spectrum Disorder[Title/Abstract] OR ASD[Title/Abstract] OR Asperger[Title/Abstract] OR Mental Retardation[Title/Abstract] OR Intellectual Disability[Title/Abstract] OR Speech[Title/Abstract] OR Language[Title/Abstract] OR Asperger[Title/Abstract] OR Mutism[Title/Abstract] OR Emotional Disorder[Title/Abstract] OR Reactive Attachment [Title/Abstract] OR Suicide*[Title/Abstract] OR Attention Deficit Hyperactivity Disorder[Title/Abstract] OR ADHD[Title/Abstract] OR Hyperkinetic[Title/Abstract] OR Eating Disorder[Title/Abstract] OR Anorexi*[Title/Abstract] OR Bulimi*[Title/Abstract] OR Dement*[Title/Abstract] OR Amnesi*[Title/Abstract] OR Deliri*[Title/Abstract] OR Halluci*[Title/Abstract] OR Cataton*[Title/Abstract] OR Labil*[Title/Abstract] OR Cogniti*[Title/Abstract] OR Epilep*[Title/Abstract] OR Personality Disorder[Title/Abstract] OR Personality[Title/Abstract] OR PD[Title/Abstract]) | 3832356 |
| #6 | #4 AND #5 | 2333 |

**Table A.2 Embase**

| No. | Searches | Results |
| --- | --- | --- |
| #1 | 'covid 19':ab,ti OR covid19:ab,ti OR '2019 novel coronavirus infection':ab,ti OR '2019-ncov infection':ab,ti OR 'covid-19 pandemic':ab,ti OR 'coronavirus disease-19':ab,ti OR '2019-ncov disease':ab,ti OR '2019 novel coronavirus disease':ab,ti OR 'coronavirus disease 2019':ab,ti OR 'severe acute respiratory syndrome coronavirus 2':ab,ti OR 'wuhan coronavirus':ab,ti OR 'wuhan seafood market pneumonia virus':ab,ti OR 'covid19 virus':ab,ti OR 'covid-19 virus':ab,ti OR 'coronavirus disease 2019 virus':ab,ti OR 'sars cov 2':ab,ti OR sars2:ab,ti OR '2019 ncov':ab,ti OR '2019 novel coronavirus':ab,ti OR  'severe acute respiratory syndrome'/exp OR ‘SARS coronavirus’/exp OR ‘sars’:ab,ti OR 'severe acute respiratory syndrome virus':ab,ti OR 'sars-related coronavirus':ab,ti OR 'coronavirus, sars-related':ab,ti OR 'sars related coronavirus':ab,ti OR 'sars cov':ab,ti OR 'urbani sars-associated coronavirus':ab,ti OR 'coronavirus, urbani sars-associated':ab,ti OR 'sars-associated coronavirus, urbani':ab,ti OR 'urbani sars associated coronavirus':ab,ti OR 'sars coronavirus':ab,ti OR 'coronavirus, sars':ab,ti OR 'severe acute respiratory syndrome-related coronavirus':ab,ti OR 'severe acute respiratory syndrome related coronavirus':ab,ti OR 'sars-associated coronavirus':ab,ti OR 'coronavirus, sars-associated':ab,ti OR 'sars associated coronavirus':ab,ti OR  ‘Middle East respiratory syndrome coronavirus’/exp OR ‘mers’:ab,ti OR 'mers cov':ab,ti OR 'mers virus':ab,ti OR 'mers viruses':ab,ti OR 'virus, mers':ab,ti OR 'viruses, mers':ab,ti OR 'middle east respiratory syndrome-related coronavirus':ab,ti OR 'middle east respiratory syndrome related coronavirus':ab,ti | 19,268 |
| #2 | (‘influenza’/exp AND (‘epidemic’/exp OR ‘pandemic’/exp)) OR  (‘influenza’:ab,ti OR ‘influenzas’:ab,ti OR 'human influenzas':ab,ti OR 'influenzas, human':ab,ti OR 'human flu':ab,ti OR 'flu, human':ab,ti OR 'human influenza':ab,ti OR 'influenza in humans':ab,ti OR 'influenza in human':ab,ti OR grippe:ab,ti) AND ('disease outbreaks':ab,ti OR 'disease outbreak':ab,ti OR 'outbreak, disease':ab,ti OR 'outbreaks, disease':ab,ti OR outbreaks:ab,ti OR 'infectious disease outbreaks':ab,ti OR 'disease outbreak, infectious':ab,ti OR 'disease outbreaks, infectious':ab,ti OR 'infectious disease outbreak':ab,ti OR 'outbreak, infectious disease':ab,ti OR 'outbreaks, infectious disease':ab,ti OR pandemic:ab,ti OR pandemics:ab,ti OR epidemic:ab,ti OR epidemics:ab,ti) | 27,656 |
| #3 | #1 OR #2 | 46,021 |
| #4 | ‘mental health’/exp OR ‘mental disease’/exp OR  'mental health':ab,ti OR 'mental hygiene':ab,ti OR 'psychological health':ab,ti OR 'psychological hygiene':ab,ti OR 'mental disorder':ab,ti OR 'psychiatric disorder':ab,ti OR psychiatr*:ab,ti OR psychosis:ab,ti OR schizophreni*:ab,ti OR delusion*:ab,ti OR paranoi*:ab,ti OR mania:ab,ti OR manic:ab,ti OR hypomania:ab,ti OR 'bipolar disorder':ab,ti OR bipolar:ab,ti OR 'mood disorder':ab,ti OR 'mood disorders':ab,ti OR depress*:ab,ti OR 'depressive disorder':ab,ti OR 'major depressive disorder':ab,ti OR cyclothymia:ab,ti OR dysthymia:ab,ti OR phobi*:ab,ti OR fear:ab,ti OR agoraphobi*:ab,ti OR 'generalised anxiety disorder':ab,ti OR 'generalized anxiety disorder':ab,ti OR 'anxiety disorder':ab,ti OR anxiet*:ab,ti OR 'panic disorder':ab,ti OR 'panic attack':ab,ti OR panic:ab,ti OR obsessi*:ab,ti OR compulsi*:ab,ti OR 'obsessive compulsive disorder':ab,ti OR ocd:ab,ti OR stress:ab,ti OR 'post traumatic stress disorder':ab,ti OR ptsd:ab,ti OR 'adjustment disorder':ab,ti OR adjustment:ab,ti OR 'dissociative disorder':ab,ti OR dissociat*:ab,ti OR 'somatoform disorder':ab,ti OR somatoform:ab,ti OR hypochondria*:ab,ti OR depersonali*:ab,ti OR dereali*:ab,ti OR 'sleep disorder':ab,ti OR sleep:ab,ti OR insomnia:ab,ti OR hypersomnia:ab,ti OR 'sleep terror':ab,ti OR nightmare:ab,ti OR sleepwalking:ab,ti OR 'sexual dysfunction':ab,ti OR sexual:ab,ti OR 'motor function':ab,ti OR developmental:ab,ti OR autism:ab,ti OR 'autism spectrum disorder':ab,ti OR asd:ab,ti OR 'mental retardation':ab,ti OR 'intellectual disability':ab,ti OR speech:ab,ti OR language:ab,ti OR asperger:ab,ti OR mutism:ab,ti OR 'emotional disorder':ab,ti OR 'reactive attachment':ab,ti OR suicide*:ab,ti OR 'attention deficit hyperactivity disorder':ab,ti OR adhd:ab,ti OR hyperkinetic:ab,ti OR 'eating disorder':ab,ti OR anorexi*:ab,ti OR bulimi*:ab,ti OR dement*:ab,ti OR amnesi*:ab,ti OR deliri*:ab,ti OR halluci*:ab,ti OR cataton*:ab,ti OR labil*:ab,ti OR cogniti*:ab,ti OR epilep*:ab,ti OR 'personality disorder':ab,ti OR ‘personality’:ab,ti OR ‘pd’:ab,ti | 5,238,249 |
| #5 | #3 AND #4 | 3,011 |

**Table A.3 PsycINFO**

| No. | Searches | Results |
| --- | --- | --- |
| #1 | (COVID-19 or COVID19 or 2019 novel coronavirus infection or 2019-nCoV infection or COVID-19 pandemic or coronavirus disease-19 or 2019-nCoV disease or 2019 novel coronavirus disease or coronavirus disease 2019 or severe acute respiratory syndrome coronavirus 2 or Wuhan coronavirus or Wuhan seafood market pneumonia virus or COVID19 virus or COVID-19 virus or coronavirus disease 2019 virus or SARS-CoV-2 or SARS2 or 2019-nCoV or 2019 novel coronavirus or  SARS or Severe Acute Respiratory Syndrome Virus or SARS-Related Coronavirus or Coronavirus, SARS-Related or SARS Related Coronavirus or SARS-CoV or Urbani SARS-Associated Coronavirus or Coronavirus, Urbani SARS-Associated or SARS-Associated Coronavirus, Urbani or Urbani SARS Associated Coronavirus or SARS Coronavirus or Coronavirus, SARS or Severe acute respiratory syndrome-related coronavirus or Severe acute respiratory syndrome related coronavirus or SARS-Associated Coronavirus or Coronavirus, SARS-Associated or SARS Associated Coronavirus or  MERS or MERS-CoV or MERS Virus or MERS Viruses or Virus, MERS or Viruses, MERS or Middle East respiratory syndrome-related coronavirus or Middle East respiratory syndrome related coronavirus).ti,ab. | 539 |
| #2 | Exp Influenza/ AND (exp epidemics/ or exp pandemic/) | 274 |
| #3 | (Influenza or Influenzas or Human Influenzas or Influenzas, Human or Human Flu or Flu, Human or Human Influenza or Influenza in Humans or Influenza in Human or Grippe).ti,ab.  AND  (Disease Outbreaks or Disease Outbreak or Outbreak, Disease or Outbreaks, Disease or Outbreaks or Infectious Disease Outbreaks or Disease Outbreak, Infectious or Disease Outbreaks, Infectious or Infectious Disease Outbreak or Outbreak, Infectious Disease or Outbreaks, Infectious Disease or Pandemic or Pandemics or Epidemic or Epidemics).ti,ab. | 682 |
| #4 | 2 or 3 | 713 |
| #5 | 1 or 4 | 1215 |
| #6 | Exp Mental health/ or exp mental disorders/ or  (Mental Health or Mental Hygiene or Psychological Health or Psychological Hygiene or Mental Disorder or Psychiatric Disorder or Psychiatr* or Psychosis or Schizophreni* or Delusion* or Paranoi* or Mania or Manic or Hypomania or Bipolar Disorder or Bipolar or Mood Disorder or Mood Disorders or Depress* or Depressive Disorder or Major Depressive Disorder or Cyclothymia or Dysthymia or Phobi* or Fear or Agoraphobi* or Generalised Anxiety Disorder or Generalized Anxiety Disorder or Anxiety Disorder or Anxiet* or Panic Disorder or Panic Attack or Panic or Obsessi* or Compulsi* or Obsessive Compulsive Disorder or OCD or Stress or Post Traumatic Stress Disorder or PTSD or Adjustment Disorder or Adjustment or Dissociative Disorder or Dissociat* or Somatoform Disorder or Somatoform or Hypochondria* or Depersonali* or Dereali* or Sleep Disorder or Sleep or Insomnia or Hypersomnia or Sleep Terror or Nightmare or Sleepwalking or Sexual Dysfunction or Sexual or Motor Function or Developmental or Autism or Autism Spectrum Disorder or ASD or Asperger or Mental Retardation or Intellectual Disability or Speech or Language or Asperger or Mutism or Emotional Disorder or Reactive Attachment or Suicide* or Attention Deficit Hyperactivity Disorder or ADHD or Hyperkinetic or Eating Disorder or Anorexi* or Bulimi* or Dement* or Amnesi* or Deliri* or Halluci* or Cataton* or Labil* or Cogniti* or Epilep* or Personality Disorder or Personality or PD).ti,ab. | 2,203,686 |
| #7 | #5 and #6 | 440 |

**Table A.4 CINAHL**

| No. | Searches | Results |
| --- | --- | --- |
| #1 | MH Middle East Respiratory Syndrome Coronavirus OR MH Middle East Respiratory Syndrome OR MH SARS Virus OR TI ( COVID-19 or COVID19 or 2019 novel coronavirus infection or 2019-nCoV infection or COVID-19 pandemic or coronavirus disease-19 or 2019-nCoV disease or 2019 novel coronavirus disease or coronavirus disease 2019 or severe acute respiratory syndrome coronavirus 2 or Wuhan coronavirus or Wuhan seafood market pneumonia virus or COVID19 virus or COVID-19 virus or coronavirus disease 2019 virus or SARS-CoV-2 or SARS2 or 2019-nCoV or 2019 novel coronavirus ) OR AB ( COVID-19 or COVID19 or 2019 novel coronavirus infection or 2019-nCoV infection or COVID-19 pandemic or coronavirus disease-19 or 2019-nCoV disease or 2019 novel coronavirus disease or coronavirus disease 2019 or severe acute respiratory syndrome coronavirus 2 or Wuhan coronavirus or Wuhan seafood market pneumonia virus or COVID19 virus or COVID-19 virus or coronavirus disease 2019 virus or SARS-CoV-2 or SARS2 or 2019-nCoV or 2019 novel coronavirus ) OR TI ( MERS or MERS-CoV or MERS Virus or MERS Viruses or Virus, MERS or Viruses, MERS or Middle East respiratory syndrome-related coronavirus or Middle East respiratory syndrome related coronavirus ) OR AB ( MERS or MERS-CoV or MERS Virus or MERS Viruses or Virus, MERS or Viruses, MERS or Middle East respiratory syndrome-related coronavirus or Middle East respiratory syndrome related coronavirus ) OR TI ( SARS or Severe Acute Respiratory Syndrome Virus or SARS-Related Coronavirus or Coronavirus, SARS-Related or SARS Related Coronavirus or SARS-CoV or Urbani SARS-Associated Coronavirus or Coronavirus, Urbani SARS-Associated or SARS-Associated Coronavirus, Urbani or Urbani SARS Associated Coronavirus or SARS Coronavirus or Coronavirus, SARS or Severe acute respiratory syndrome-related coronavirus or Severe acute respiratory syndrome related coronavirus or SARS-Associated Coronavirus or Coronavirus, SARS-Associated or SARS Associated Coronavirus ) OR AB ( SARS or Severe Acute Respiratory Syndrome Virus or SARS-Related Coronavirus or Coronavirus, SARS-Related or SARS Related Coronavirus or SARS-CoV or Urbani SARS-Associated Coronavirus or Coronavirus, Urbani SARS-Associated or SARS-Associated Coronavirus, Urbani or Urbani SARS Associated Coronavirus or SARS Coronavirus or Coronavirus, SARS or Severe acute respiratory syndrome-related coronavirus or Severe acute respiratory syndrome related coronavirus or SARS-Associated Coronavirus or Coronavirus, SARS-Associated or SARS Associated Coronavirus ) | 3739 |
| #2 | MH Influenza, Human AND MH Disease Outbreaks | 2016 |
| #3 | TI ( Influenza or Influenzas or Human Influenzas or Influenzas, Human or Human Flu or Flu, Human or Human Influenza or Influenza in Humans or Influenza in Human or Grippe ) OR AB ( Influenza or Influenzas or Human Influenzas or Influenzas, Human or Human Flu or Flu, Human or Human Influenza or Influenza in Humans or Influenza in Human or Grippe ) | 19,364) |
| #4 | TI ( Disease Outbreaks or Disease Outbreak or Outbreak, Disease or Outbreaks, Disease or Outbreaks or Infectious Disease Outbreaks or Disease Outbreak, Infectious or Disease Outbreaks, Infectious or Infectious Disease Outbreak or Outbreak, Infectious Disease or Outbreaks, Infectious Disease or Pandemic or Pandemics or Epidemic or Epidemics ) OR AB ( Disease Outbreaks or Disease Outbreak or Outbreak, Disease or Outbreaks, Disease or Outbreaks or Infectious Disease Outbreaks or Disease Outbreak, Infectious or Disease Outbreaks, Infectious or Infectious Disease Outbreak or Outbreak, Infectious Disease or Outbreaks, Infectious Disease or Pandemic or Pandemics or Epidemic or Epidemics ) | 42,736 |
| #5 | #3 AND #4 | 5,421 |
| #6 | #1 OR #2 OR #5 | 9787 |
| #7 | MH mental health OR MH mental disorders OR TI ( Mental Health or Mental Hygiene or Psychological Health or Psychological Hygiene or Mental Disorder or Psychiatric Disorder or Psychiatr* or Psychosis or Schizophreni* or Delusion* or Paranoi* or Mania or Manic or Hypomania or Bipolar Disorder or Bipolar or Mood Disorder or Mood Disorders or Depress* or Depressive Disorder or Major Depressive Disorder or Cyclothymia or Dysthymia or Phobi* or Fear or Agoraphobi* or Generalised Anxiety Disorder or Generalized Anxiety Disorder or Anxiety Disorder or Anxiet* or Panic Disorder or Panic Attack or Panic or Obsessi* or Compulsi* or Obsessive Compulsive Disorder or OCD or Stress or Post Traumatic Stress Disorder or PTSD or Adjustment Disorder or Adjustment or Dissociative Disorder or Dissociat* or Somatoform Disorder or Somatoform or Hypochondria* or Depersonali* or Dereali* or Sleep Disorder or Sleep or Insomnia or Hypersomnia or Sleep Terror or Nightmare or Sleepwalking or Sexual Dysfunction or Sexual or Motor Function or Developmental or Autism or Autism Spectrum Disorder or ASD or Asperger or Mental Retardation or Intellectual Disability or Speech or Language or Asperger or Mutism or Emotional Disorder or Reactive Attachment or Suicide* or Attention Deficit Hyperactivity Disorder or ADHD or Hyperkinetic or Eating Disorder or Anorexi* or Bulimi* or Dement* or Amnesi* or Deliri* or Halluci* or Cataton* or Labil* or Cogniti* or Epilep* or Personality Disorder or Personality or PD ) OR AB ( Mental Health or Mental Hygiene or Psychological Health or Psychological Hygiene or Mental Disorder or Psychiatric Disorder or Psychiatr* or Psychosis or Schizophreni* or Delusion* or Paranoi* or Mania or Manic or Hypomania or Bipolar Disorder or Bipolar or Mood Disorder or Mood Disorders or Depress* or Depressive Disorder or Major Depressive Disorder or Cyclothymia or Dysthymia or Phobi* or Fear or Agoraphobi* or Generalised Anxiety Disorder or Generalized Anxiety Disorder or Anxiety Disorder or Anxiet* or Panic Disorder or Panic Attack or Panic or Obsessi* or Compulsi* or Obsessive Compulsive Disorder or OCD or Stress or Post Traumatic Stress Disorder or PTSD or Adjustment Disorder or Adjustment or Dissociative Disorder or Dissociat* or Somatoform Disorder or Somatoform or Hypochondria* or Depersonali* or Dereali* or Sleep Disorder or Sleep or Insomnia or Hypersomnia or Sleep Terror or Nightmare or Sleepwalking or Sexual Dysfunction or Sexual or Motor Function or Developmental or Autism or Autism Spectrum Disorder or ASD or Asperger or Mental Retardation or Intellectual Disability or Speech or Language or Asperger or Mutism or Emotional Disorder or Reactive Attachment or Suicide* or Attention Deficit Hyperactivity Disorder or ADHD or Hyperkinetic or Eating Disorder or Anorexi* or Bulimi* or Dement* or Amnesi* or Deliri* or Halluci* or Cataton* or Labil* or Cogniti* or Epilep* or Personality Disorder or Personality or PD ) | 1,081,161 |
| #8 | #6 AND #7 | 722 |

**Table A.5 Scopus**

| No. | Searches | Results |
| --- | --- | --- |
| #1 | TITLE-ABS-KEY ( ( covid-19 ) OR ( covid19 ) OR ( 2019 novel AND coronavirus AND infection ) OR ( 2019-ncov AND infection ) OR ( covid-19 AND pandemic ) OR ( coronavirus AND disease-19 ) OR ( 2019-ncov AND disease ) OR ( 2019 novel AND coronavirus AND disease ) OR ( coronavirus AND disease 2019 ) OR ( severe AND acute AND respiratory AND syndrome AND coronavirus 2 ) OR ( wuhan AND coronavirus ) OR ( wuhan AND seafood AND market AND pneumonia AND virus ) OR ( covid19 AND virus ) OR ( covid-19 AND virus ) OR ( coronavirus AND disease 2019 virus ) OR ( sars-cov-2 ) OR ( sars2 ) OR ( 2019-ncov ) OR ( 2019 novel AND coronavirus ) OR ( mers ) OR ( mers-cov ) OR ( mers AND virus ) OR ( mers AND viruses ) OR ( virus, AND mers ) OR ( viruses, AND mers ) OR ( middle AND east AND respiratory AND syndrome-related AND coronavirus ) OR ( middle AND east AND respiratory AND syndrome AND related AND coronavirus ) OR ( sars ) OR ( severe AND acute AND respiratory AND syndrome AND virus ) OR ( sars-related AND coronavirus ) OR ( coronavirus, AND sars-related ) OR ( sars AND related AND coronavirus ) OR ( sars-cov ) OR ( urbani AND sars-associated AND coronavirus ) OR ( coronavirus, AND urbani AND sars-associated ) OR ( sars-associated AND coronavirus, AND urbani ) OR ( urbani AND sars AND associated AND coronavirus ) OR ( sars AND coronavirus ) OR ( coronavirus, AND sars ) OR ( severe AND acute AND respiratory AND syndrome-related AND coronavirus ) OR ( severe AND acute AND respiratory AND syndrome AND related AND coronavirus ) OR ( sars-associated AND coronavirus ) OR ( coronavirus, AND sars-associated ) OR ( sars AND associated AND coronavirus ) ) | 136,309 |
| #2 | TITLE-ABS-KEY ( ( ( influenza ) OR ( influenzas ) OR ( human AND influenzas ) OR ( influenzas, AND human ) OR ( human AND flu ) OR ( flu, AND human ) OR ( human AND influenza ) OR ( influenza AND in AND humans ) OR ( influenza AND in AND human ) OR ( grippe ) ) AND ( ( disease AND outbreaks ) OR ( disease AND outbreak ) OR ( outbreak, AND disease ) OR ( outbreaks, AND disease ) OR ( outbreaks ) OR ( infectious AND disease AND outbreaks ) OR ( disease AND outbreak, AND infectious ) OR ( disease AND outbreaks, AND infectious ) OR ( infectious AND disease AND outbreak ) OR ( outbreak, AND infectious AND disease ) OR ( outbreaks, AND infectious AND disease ) OR ( pandemic ) OR ( pandemics ) OR ( epidemic ) OR ( epidemics ) ) ) | 38,362 |
| #3 | #1 or #2 | 172,671 |
| #4 | TITLE-ABS-KEY ( ( mental AND health ) OR ( mental AND hygiene ) OR ( psychological AND health ) OR ( psychological AND hygiene ) OR ( mental AND disorder ) OR ( psychiatric AND disorder ) OR ( psychiatr* ) OR ( psychosis ) OR ( schizophreni* ) OR ( delusion* ) OR ( paranoi* ) OR ( mania ) OR ( manic ) OR ( hypomania ) OR ( bipolar AND disorder ) OR ( bipolar ) OR ( mood AND disorder ) OR ( mood AND disorders ) OR ( depress* ) OR ( depressive AND disorder ) OR ( major AND depressive AND disorder ) OR ( cyclothymia ) OR ( dysthymia ) OR ( phobi* ) OR ( fear ) OR ( agoraphobi* ) OR ( generalised AND anxiety AND disorder ) OR ( generalized AND anxiety AND disorder ) OR ( anxiety AND disorder ) OR ( anxiet* ) OR ( panic AND disorder ) OR ( panic AND attack ) OR ( panic ) OR ( obsessi* ) OR ( compulsi* ) OR ( obsessive AND compulsive AND disorder ) OR ( ocd ) OR ( stress ) OR ( post AND traumatic AND stress AND disorder ) OR ( ptsd ) OR ( adjustment AND disorder ) OR ( adjustment ) OR ( dissociative AND disorder ) OR ( dissociat* ) OR ( somatoform AND disorder ) OR ( somatoform ) OR ( hypochondria* ) OR ( depersonali* ) OR ( dereali* ) OR ( sleep AND disorder ) OR ( sleep ) OR ( insomnia ) OR ( hypersomnia ) OR ( sleep AND terror ) OR ( nightmare ) OR ( sleepwalking ) OR ( sexual AND dysfunction ) OR ( sexual ) OR ( motor AND function ) OR ( developmental ) OR ( autism ) OR ( autism AND spectrum AND disorder ) OR ( asd ) OR ( asperger ) OR ( mental AND retardation ) OR ( intellectual AND disability ) OR ( speech ) OR ( language ) OR ( asperger ) OR ( mutism ) OR ( emotional AND disorder ) OR ( reactive AND attachment ) OR ( suicide* ) OR ( attention AND deficit AND hyperactivity AND disorder ) OR ( adhd ) OR ( hyperkinetic ) OR ( eating AND disorder ) OR ( anorexi* ) OR ( bulimi* ) OR ( dement* ) OR ( amnesi* ) OR ( deliri* ) OR ( halluci* ) OR ( cataton* ) OR ( labil* ) OR ( cogniti* ) OR ( epilep* ) OR ( personality AND disorder ) OR ( personality ) OR ( pd ) ) | 8,903,103 |
| #5 | #3 AND #4 | 11,515 |
| #6 | #5 AND ( LIMIT-TO ( LANGUAGE,"English" ) ) AND ( EXCLUDE ( DOCTYPE,"ch" ) OR EXCLUDE ( DOCTYPE,"no" ) OR EXCLUDE ( DOCTYPE,"ed" ) OR EXCLUDE ( DOCTYPE,"le" ) OR EXCLUDE ( DOCTYPE,"sh" ) OR EXCLUDE ( DOCTYPE,"bk" ) OR EXCLUDE ( DOCTYPE,"cr" ) OR EXCLUDE ( DOCTYPE,"er" ) OR EXCLUDE ( DOCTYPE,"bz" ) ) AND ( EXCLUDE ( EXACTKEYWORD,"Nonhuman" ) ) AND ( LIMIT-TO ( SUBJAREA,"MEDI" ) OR LIMIT-TO ( SUBJAREA,"SOCI" ) OR LIMIT-TO ( SUBJAREA,"NEUR" ) OR LIMIT-TO ( SUBJAREA,"NURS" ) OR LIMIT-TO ( SUBJAREA,"MULT" ) OR LIMIT-TO ( SUBJAREA,"PSYC" ) OR LIMIT-TO ( SUBJAREA,"HEAL" ) OR LIMIT-TO ( SUBJAREA,"DENT" ) OR LIMIT-TO ( SUBJAREA,"Undefined" ) ) | 3,317 |

**Table A.6 Web of Science**

| No. | Searches | Results |
| --- | --- | --- |
| #1 | TOPIC: (COVID-19 or COVID19 or 2019 novel coronavirus infection or 2019-nCoV infection or COVID-19 pandemic or coronavirus disease-19 or 2019-nCoV disease or 2019 novel coronavirus disease or coronavirus disease 2019 or severe acute respiratory syndrome coronavirus 2 or Wuhan coronavirus or Wuhan seafood market pneumonia virus or COVID19 virus or COVID-19 virus or coronavirus disease 2019 virus or SARS-CoV-2 or SARS2 or 2019-nCoV or 2019 novel coronavirus or SARS or Severe Acute Respiratory Syndrome Virus or SARS-Related Coronavirus or Coronavirus, SARS-Related or SARS Related Coronavirus or SARS-CoV or Urbani SARS-Associated Coronavirus or Coronavirus, Urbani SARS-Associated or SARS-Associated Coronavirus, Urbani or Urbani SARS Associated Coronavirus or SARS Coronavirus or Coronavirus, SARS or Severe acute respiratory syndrome-related coronavirus or Severe acute respiratory syndrome related coronavirus or SARS-Associated Coronavirus or Coronavirus, SARS-Associated or SARS Associated Coronavirus or MERS or MERS-CoV or MERS Virus or MERS Viruses or Virus, MERS or Viruses, MERS or Middle East respiratory syndrome-related coronavirus or Middle East respiratory syndrome related coronavirus)  Indexes=SCI-EXPANDED, SSCI, A&HCI, CPCI-S, CPCI-SSH, ESCI Timespan=All years | 38,538 |
| #2 | TOPIC: ((Influenza or Influenzas or Human Influenzas or Influenzas, Human or Human Flu or Flu, Human or Human Influenza or Influenza in Humans or Influenza in Human or Grippe) AND (Disease Outbreaks or Disease Outbreak or Outbreak, Disease or Outbreaks, Disease or Outbreaks or Infectious Disease Outbreaks or Disease Outbreak, Infectious or Disease Outbreaks, Infectious or Infectious Disease Outbreak or Outbreak, Infectious Disease or Outbreaks, Infectious Disease or Pandemic or Pandemics or Epidemic or Epidemics))  Indexes=SCI-EXPANDED, SSCI, A&HCI, CPCI-S, CPCI-SSH, ESCI Timespan=All years | 28,681 |
| #3 | #1 or #2 | 65,976 |
| #4 | TOPIC: (Mental Health or Mental Hygiene or Psychological Health or Psychological Hygiene or Mental Disorder or Psychiatric Disorder or Psychiatr* or Psychosis or Schizophreni* or Delusion* or Paranoi* or Mania or Manic or Hypomania or Bipolar Disorder or Bipolar or Mood Disorder or Mood Disorders or Depress* or Depressive Disorder or Major Depressive Disorder or Cyclothymia or Dysthymia or Phobi* or Fear or Agoraphobi* or Generalised Anxiety Disorder or Generalized Anxiety Disorder or Anxiety Disorder or Anxiet* or Panic Disorder or Panic Attack or Panic or Obsessi* or Compulsi* or Obsessive Compulsive Disorder or OCD or Stress or Post Traumatic Stress Disorder or PTSD or Adjustment Disorder or Adjustment or Dissociative Disorder or Dissociat* or Somatoform Disorder or Somatoform or Hypochondria* or Depersonali* or Dereali* or Sleep Disorder or Sleep or Insomnia or Hypersomnia or Sleep Terror or Nightmare or Sleepwalking or Sexual Dysfunction or Sexual or Motor Function or Developmental or Autism or Autism Spectrum Disorder or ASD or Asperger or Mental Retardation or Intellectual Disability or Speech or Language or Asperger or Mutism or Emotional Disorder or Reactive Attachment or Suicide* or Attention Deficit Hyperactivity Disorder or ADHD or Hyperkinetic or Eating Disorder or Anorexi* or Bulimi* or Dement* or Amnesi* or Deliri* or Halluci* or Cataton* or Labil* or Cogniti* or Epilep* or Personality Disorder or Personality or PD)  Indexes=SCI-EXPANDED, SSCI, A&HCI, CPCI-S, CPCI-SSH, ESCI Timespan=All years | 6,683,703 |
| #5 | #3 and #4 | 4,938 |

**Table A.7 Cochrane**

| No. | Searches | Results |
| --- | --- | --- |
| #1 | (COVID-19 or COVID19 or 2019 novel coronavirus infection or 2019 nCoV infection or COVID-19 pandemic or coronavirus disease-19 or 2019 nCoV disease or 2019 novel coronavirus disease or coronavirus disease 2019 or severe acute respiratory syndrome coronavirus 2 or Wuhan coronavirus or Wuhan seafood market pneumonia virus or COVID19 virus or COVID-19 virus or coronavirus disease 2019 virus or SARS-CoV-2 or SARS2 or 2019 nCoV or 2019 novel coronavirus or SARS or Severe Acute Respiratory Syndrome Virus or SARS-Related Coronavirus or Coronavirus, SARS-Related or SARS Related Coronavirus or SARS-CoV or Urbani SARS-Associated Coronavirus or Coronavirus, Urbani SARS-Associated or SARS-Associated Coronavirus, Urbani or Urbani SARS Associated Coronavirus or SARS Coronavirus or Coronavirus, SARS or Severe acute respiratory syndrome-related coronavirus or Severe acute respiratory syndrome related coronavirus or SARS-Associated Coronavirus or Coronavirus, SARS-Associated or SARS Associated Coronavirus or MERS or MERS-CoV or MERS Virus or MERS Viruses or Virus, MERS or Viruses, MERS or Middle East respiratory syndrome-related coronavirus or Middle East respiratory syndrome related coronavirus):ti,ab,kw | 1218 |
| #2 | ((Influenza or Influenzas or Human Influenzas or Influenzas, Human or Human Flu or Flu, Human or Human Influenza or Influenza in Humans or Influenza in Human or Grippe) AND (Disease Outbreaks or Disease Outbreak or Outbreak, Disease or Outbreaks, Disease or Outbreaks or Infectious Disease Outbreaks or Disease Outbreak, Infectious or Disease Outbreaks, Infectious or Infectious Disease Outbreak or Outbreak, Infectious Disease or Outbreaks, Infectious Disease or Pandemic or Pandemics or Epidemic or Epidemics)):ti,ab,kw | 1016 |
| #3 | #1 or #2 | 2212 |
| #4 | (Mental Health or Mental Hygiene or Psychological Health or Psychological Hygiene or Mental Disorder or Psychiatric Disorder or Psychiatr* or Psychosis or Schizophreni* or Delusion* or Paranoi* or Mania or Manic or Hypomania or Bipolar Disorder or Bipolar or Mood Disorder or Mood Disorders or Depress* or Depressive Disorder or Major Depressive Disorder or Cyclothymia or Dysthymia or Phobi* or Fear or Agoraphobi* or Generalised Anxiety Disorder or Generalized Anxiety Disorder or Anxiety Disorder or Anxiet* or Panic Disorder or Panic Attack or Panic or Obsessi* or Compulsi* or Obsessive Compulsive Disorder or OCD or Stress or Post Traumatic Stress Disorder or PTSD or Adjustment Disorder or Adjustment or Dissociative Disorder or Dissociat* or Somatoform Disorder or Somatoform or Hypochondria* or Depersonali* or Dereali* or Sleep Disorder or Sleep or Insomnia or Hypersomnia or Sleep Terror or Nightmare or Sleepwalking or Sexual Dysfunction or Sexual or Motor Function or Developmental or Autism or Autism Spectrum Disorder or ASD or Asperger or Mental Retardation or Intellectual Disability or Speech or Language or Asperger or Mutism or Emotional Disorder or Reactive Attachment or Suicide* or Attention Deficit Hyperactivity Disorder or ADHD or Hyperkinetic or Eating Disorder or Anorexi* or Bulimi* or Dement* or Amnesi* or Deliri* or Halluci* or Cataton* or Labil* or Cogniti* or Epilep* or Personality Disorder or Personality or PD):ti,ab,kw | 444,492 |
| #5 | #3 and #4 | 506 |

**Appendix B Study characteristics and summary of results**

**Table B.1 Summaries of studies (n = 30) for mental health impact on general population**

| **Author (year)** | **Country** | **Subgroup** | **Respiratory epidemics** | **Reported outcome** | **Timeframe** | **Study design** | **Data collection** | **Sample size** | **Scale used** | **Results** |
| --- | --- | --- | --- | --- | --- | --- | --- | --- | --- | --- |
| Al-Rabiaah (2020) | Saudi Arabia | Healthcare students | MERS | Anxiety | During | Cross-sectional | Online questionnaire | 174 | Generalised Anxiety Disorder-7 criteria (GAD-7) | GAD score is associated with perceived social avoidance score and perceived change hygienic habits score (hand washing, buying more sanitizers, compliance with universal precautions, avoiding contact with people with flu symptoms, avoiding social gatherings, handshaking, and public utilities) |
| Chan (2006) | Hong Kong | Elderly aged 65 and over | SARS | Suicide | During | Cross-sectional | Government statistics | NA | Government statistics | The excess elderly suicide deaths in 2003 were exclusive to April 2003, which coincided with the majority of the SARS incident cases. Female gender has higher risk. |
| Cheng (2005) | Hong Kong | General undergraduates | SARS | Anxiety | During and after | Prospective cohort | Online questionnaire | 72 | State-Trait Anxiety Inventory (STAI) | Fluctuations in state anxiety across time points. Results from hierarchical linear modeling showed that trait anxiety as well as the situation‐appropriate coping strategies of avoidance and personal hygiene practice accounted for changes in state anxiety. |
| Cheung (2008) | Hong Kong | Elderly aged 65 and over | SARS | Suicide | During and after | Cross-sectional | Coroners' court reporting | NA | Government statistics | Worrying or fearing of contracting the disease was one of the common characteristics of older adults who committed suicide during the peri-SARS period. In the pre-SARS and peri-SARS periods, there were more old people with terminal or severe illness who commit suicide. Those who are totally or partially dependent were over-represented in the suicide cases in the pre-SARS and peri-SARS period. The proportion of older adults who were worrying of having sickness was substantially larger in the peri-SARS period. |
| Cowling (2010) | Hong Kong | General public | Influenza A/H1N1 | Anxiety | During | Cross-sectional | Telephone survey | 12965 | State Trait Anxiety Inventory (STAI) | Anxiety in public is generally low. Perceived severity compared with SARS was initially very high but declined sharply during May and remained stable at a lower level through the remainder of the epidemic. Higher anxiety is associated with greater social distancing, lower cough etiquette scores and lower use of all 4 hand hygiene measures. |
| Elizarrarás-Rivas (2010) | Mexico | Family members of patients | Influenza A/H1N1 | Depression, Anxiety | During | Cross-sectional | Self-reported questionnaire | 35 | Center for Epidemiologic Studies Depression Scale (CES-D), Death Anxiety Questionnaire (DAQ) | 43% above score 16 which is significant for risk of clinical depression. Factors associated with depression include female, non-spousal family relationships and increasing age in years. Factors associated with death anxiety (attitudes towards one's own death and dying, including fear of unknown, suffering, loneliness, and personal extinction) includes increasing age in years and university-level education. |
| Kang (2018) | South Korea | General public | MERS | Anxiety | After | Cross-sectional | Online questionnaire | 400 | Modified scale from Dillard et al. (1996) and Dillard and Anderson (2004) | Higher anxiety is associated with the closer a respondent lived to the quarantined hospitals, high level of distrust in government and high uncertainty |
| Ko (2006) | Taiwan | General public | SARS | Depression | During | Cross-sectional | Telephone survey | 1472 | Taiwanese Depression Questionnaire (TDQ) | The ‘impacted group’ had higher depressive levels. The poorer self-perceived health and economic impact factors were associated with depression. Respondents were grouped as ‘impacted group’ and ‘non-impacted group’ according to whether they or their friends and family had been quarantined, or suspected of being infected. |
| Lau (2006) | Hong Kong | General public | SARS | PTSD, Psychological distress | During | Cross-sectional | Telephone survey | 818 | Impact of event scale (IES) | Higher IES scores are high in those with lower education levels. Negative feelings (horrified, apprehensive, helpless) are more likely in females, older individuals and less educated |
| Lee TM (2006) | Hong Kong | Older adults (age unspecified) | SARS | Depression, PTSD | After | Cross-sectional | Researcher administered questionnaire |  | Chinese Version: Center for Epidemiological Studies of Depression (CES-D), Impact of Event Scale-Revised (IES-R) | Those in the older-aged group (≥60yo) had significantly higher CES-D scores than those in the middle-aged group (35-59yo). After controlling for the level of depression, statistically significant main effects were obtained for location across the CIES-R scale and its three subscales. The residents in high SARS-prevalent regions consistently developed more intense symptoms of intrusion, avoidance, and hyperarousal, and a greater degree of overall post-traumatic disturbance, than the residents in areas relatively free of SARS. For cases of probable PTSD, both older age and residence in a SARS- prevalent region were associated with more clinically significant PTSD cases. |
| Lee DT (2006) | Hong Kong | Pregnant women | SARS | Depression (antenatal), Anxiety | During | Case-control | Semi-structured interview and self-reported questionnaire | Pre-SARS cohort: n=939  SARS cohort: n=235 | Beck Depression Inventory (BDI), State-Trait Anxiety Inventory (STAI) | The SARS cohort had significantly higher anxiety state scores than the pre-SARS group. Lack of social support is correlated with BDI score. BDI scores and rates of probable depression (BDI>14.5) not significant between SARS and pre-SARS groups. |
| Lee (2018) | South Korea | Patients requiring hemodialysis | MERS | Depression, Anxiety | During | Cross-sectional | Self-reported questionnaire and telephone survey | 73 | Mini International Neuropsychiatric Interview (MINI), Hospital Anxiety and Depression Scale (HADS) | The proportion of depression assessed using an abbreviated MINI was 5.5%, while when it was assessed using HADS was 15.1%. This is lower than the general population depression rate of CKD patients (20% reported by a meta-analysis). Patients received psychological support by hospital psychiatrists upon start of quarantine |
| Leung (2004) | Hong Kong and Singapore | General public | SARS | Anxiety | During | Cross-sectional | Telephone survey | Hong Kong: n=705  Singapore: n=1201 | State Trait Anxiety Inventory (STAI) | Level of anxiety demonstrated a positive dose-response gradient with the adoption of personal protective measures. Hong Kong respondents had significantly higher anxiety than Singapore respondents. Hong Kong had experienced a much more dramatic outbreak with at least three superspreading events compared with Singapore. |
| Li (2020) | China | General public, HCW | COVID-19 | Vicarious traumatisation | During | Cross-sectional | Self-reported questionnaire | 214 | Vicarious Trauma Scale | The general public and medical staff suffer from vicarious traumatization. However, the vicarious traumatization of non-front-line medical staff is more serious than that of front-line medical staff. |
| Liu (2020) | China | General public | COVID-19 | PTSD | During | Cross-sectional | Self-reported questionnaire | 285 | PTSD Checklist for DSM-5 (PCL-5), Pittsburgh Sleep Quality Index (PSQI) | 2019-Cov pandemics have a high prevalence of PTSS in the hardest-hit areas in China of 7%. Most importantly, PTSS sub-symptoms, including re-experiencing, negative alterations in cognition or mood and hyper-arousal, are more common in females than males. Better sleep quality and unfragmented sleep patterns are associated with lower PTSS prevalence. 2019-Cov pandemics have a high prevalence of PTSS in the hardest-hit areas in China of 7%. |
| Ng (2006) | Hong Kong | People with chronic diseases | SARS | Depression, Anxiety, Somatisation | During | Case-control | Self-reported questionnaire | 51  Control: n= 26  Intervention: n=25 | Brief Symptom Inventory (BSI) | Significant changes after intervention of SMART group debriefing. Depression scores dropped significantly. Anxiety, somatisation and hostility scores did not change significantly. |
| Peng (2010) | Taiwan | General public | SARS | Psychological distress | After | Cross-sectional | Telephone survey | 1278 | Brief Symptom Rating Scale | High BSRS score (>4/5) is associated with being age 50-59 years, 60 or above, a high-school graduate and worried about the recurrence of SARS. |
| Qiu (2020) | China, Hong Kong, Taiwan | General public | COVID-19 | Psychological distress | During | Cross-sectional | Online questionnaire | 52730 | COVID-19 Peritraumatic Distress Index (CPDI) | Multinomial logistic regression analyses showed that one’s CPDI score was associated with female gender, higher education, migrant workers and staying in the middle region of China (most affected by epidemic). Lower psychological distress levels are associated with male gender, availability of local medical resources, efficiency of the regional public health system, and prevention and control measures taken against the epidemic situation, age under 18 years. |
| Quah (2004) | Singapore | General public | SARS | Anxiety | During | Cross-sectional | Telephone survey | 1201 | B.A. Thyer’s Clinical Anxiety Scale | Only 2.9% of respondents reported high anxiety; mean score 3.23 S.D. of 0.48. However, the paper did not define what is the cut off score for high anxiety. Higher anxiety groups followed more preventive measures. Few reported health complaints; though reporting health complaints not is not associated with taking precautions against SARS (probably because these symptoms are related to other common diseases in SG e.g. dengue fever). Anxiety is not associated with perceived likelihood of contracting SARS. Low percentage of respondents viewed SARS as a personal risk and HCW were amongst the first SARS patients. |
| Rubin (2009) | United Kingdom | General public | Influenza A/H1N1 | Anxiety | During | Cross-sectional | Telephone survey | 1000 | State Trait Anxiety Inventory (STAI) | 23.8% (score 12 or more) has anxiety about swine flu while 2.1% (score 18 or more) has high anxiety. Participants who had carried out one or more recommended behaviours had significantly higher levels of anxiety. Participants who had carried out one or more avoidance behaviours had significantly higher levels of anxiety. Government's leaflet about swine flu reduces anxiety significantly in those who read it, compared to those who had not read it or received it. |
| Sim (2010) | Singapore | General public | SARS | PTSD | After | Cross-sectional | Self-reported questionnaire | 415 | General Health Questionnaire-8 (GHQ-8), Impact of Events Scale-Revised (IES-R) | Significant rates of SARS-related psychiatric and posttraumatic morbidities (22.9% and 25.8% respectively) in the subjects visiting our community primary health care centers, about 16 weeks after the first local outbreak of SARS. The presence of psychiatric morbidity was associated with a high level of posttraumatic symptoms. Psychiatric morbidity was further associated with being seen at fever stations, younger age, increased self blame, and less substance use, and posttraumatic morbidity was associated with increased use of denial and planning as coping measures. |
| Sprang (2013) | United States | Children and parents | Influenza A/H1N1 | PTSD | After | Cross-sectional | Focus group and questionnaire | 398 | PTSD Checklist for DSM-5 (PCL-5) | Of parents who experienced quarantine or isolation, 25% had a PTSD screening score of 25 or greater, indicating that they were at risk for PTSD; 28% had scores of 30 or greater, meeting the diagnostic criteria for PTSD. Only 7% of the parents who did not experience social distancing through isolation or quarantine had a severity score of 25 or greater, and only 5.8% scored above 30. Traumatic stress in children was measured by parent reporting on the PTSD-RI, and significant differences were also found between those who experienced social distancing measures and those who did not. Children who experienced isolation or quarantine were more likely to meet the clinical cutoff score for PTSD (30%) than those who had not been in isolation or quarantine. |
| Wan (2004) | Hong Kong | Patients (Thoracic surgery) | SARS | Depression, Anxiety | During | Cross-sectional | Telephone survey | 57 | Hospital Anxiety and Depression Scale (HADS) | 29.82% scored >15 indicating significant psychiatric problems. 42.11% have a high level of anxiety with HADS anxiety subscale score >5. Depression risk is 26.3%, with HADS depression subscale score >8. There is no relationship between waiting time on the list and level of depression and anxiety. There is no gender differences as well. |
| Wang C (2020) | China | General public | COVID-19 | Depression, Anxiety, PTSD | During | Cross-sectional | Online questionnaire | 1210 | Impact of Event Scale-Revised (IES-R), Depression Anxiety Stress Scale (DASS) | Higher IES-R and DASS scores are associated with female gender, student status, specific physical symptoms and no confidence in their own doctor’s ability to diagnose or recognize COVID-19. Higher IES-R scores are associated with high levels of concern about other family members getting COVID-19 and dissatisfaction with the amount of health information available about COVID-19. Higher DASS depression subscale scores are associated with male gender, uneducated status and breathing difficulty. Higher DASS anxiety subscale scores are associated with male gender, clinic consultations and hospitalizations, contact with an individual with suspected COVID-19 or infected materials, breathing difficulty and high levels of concern about other family members getting COVID-19. Higher DASS stress subscale scores are associated with male gender, a low perceived likelihood of surviving COVID-19 if infected, high levels of concern about other family members getting COVID-19 and dissatisfaction with the amount of health information available about COVID-19.  Lower IES-R and DASS scores are associated with specific up-to-date and accurate health information and particular precautionary measures. Lower IES-R scores are associated with male gender. Lower DASS depression subscale scores are associated with additional information on availability and effectiveness of medicines/vaccines. Lower DASS anxiety subscale scores are associated with low perceived likelihood of contracting COVID-19, regular updates for the latest information and additional information on the availability and effectiveness of medicines/vaccines. Lower DASS stress subscale scores are associated with low perceived likelihood of contracting COVID-19 and the information on the increase in the number of recovered individuals. |
| Wang Y (2020) | China | General public | COVID-19 | Depression, Anxiety | During | Cross-sectional | Online questionnaire | 600 | Self-Rating Anxiety Scale (SAS), Self-Rating Depression Scale (SDS) | SAS and SDS standard scores showed a significant positive correlation. High risk in female gender, 40 and below age group, those with a master’s degree or above (compared to those with a bachelor’s degree), professionals (compared to industrial service workers and other staff). |
| Wheaton (2011) | United States | General undergraduates | Influenza A/H1N1 | Depression, Anxiety | During | Cross-sectional | Online questionnaire | 315 | Anxiety Sensitivity Index-3, DASS, SHAI | Regression analysis indicated that only health anxiety, contamination cognitions, and disgust sensitivity, emerged as significant individual predictors. The measures of stress, body vigilance and physical anxiety sensitivity were not significant predictors of swine flu anxiety in the regression, indicating that the significant zero-order associations between these variables and swine flu anxiety are better accounted for by health anxiety, contamination cognitions, and disgust sensitivity. |
| Wong (2007) | Hong Kong | Healthcare students and general undergraduates | SARS | Anxiety | During | Cross-sectional | Online questionnaire | 763 | Zung’s Self-rating Anxiety Scale | The anxiety level caused by SARS in medical students at the teaching hospital was higher than in non-medical students in the same university and lowest in students of the other university situated 20 km away from the affected hospital. |
| Xiao (2020) | China | Self-isolated public | COVID-19 | Anxiety, Sleep | During | Cross-sectional | Self-reported questionnaire | 170 | Self-Rating Anxiety Scale (SAS), Pittsburgh Sleep Quality Index (PSQI) | Low level of social capital is associated with higher levels of anxiety. Anxiety is associated with stress and lower sleep quality. High level of social capital associated with higher level of sleep quality. With the effect of stress and anxiety, this reduces the effect of social capital on sleep quality. |
| Xu (2011) | China | General undergraduates | Influenza A/H1N1 | PTSD | After | Cross-sectional | Self-reported questionnaire | 1082 | PTSD Checklist for DSM-5 (PCL-5) | A negative relationship between the PCL-C total score and gender (female), having H1N1 influenza, having family members, friends or acquaintances having H1N1 influenza, and being afraid of H1N1 influenza was also found in this study. North China students and more academically achieving students also had higher propensity to develop PTSD. No correlation was found between PCL-C total score and having knowledge about H1N1 influenza, receiving the vaccine, and contacting people in- fected with H1N1 influenza. |
| Yu (2005) | Hong Kong | Midlife women | SARS | Depression | After | Prospective cohort | Self-reported questionnaire | 126 | Center for Epidemiologic Studies Depression Scale (CES-D) | During the SARS outbreak, a statistically significant increase in depression and perceived stress was found as indicated by CES-D and PSS mean scores. The proportion of women who scored above the cutoff of the CES-D scale increased. Although the perception of risk of SARS was not predictive of depression and stress, personal perception of risk of infection was correlated with psychological distress. Mild depression was a prevalent condition before and during SARS, highlighting the need for greater concern on women’s mental health. Emotional distress was related to risk perception and financial loss. |

**Table B.2 Summaries of studies (n = 41) for mental health impact on healthcare workers**

| **Author (year)** | **Country** | **Respiratory epidemic** | **Reported outcome** | **Timeframe** | **Study design** | **Data collection** | **Sample size** | **Scale used** | **Results** |
| --- | --- | --- | --- | --- | --- | --- | --- | --- | --- |
| Chan (2004) | Singapore | SARS | PTSD, Anxiety | After | Cross-sectional | Self-reported questionnaire | 661 | General Health Questionnaire (GHQ-28)  Impact of Event Scale (IES) | From this survey, although it was perceived that the SARS situation had greatly impacted on the emotional state of health care workers, there was no significant change in the prevalence of psychiatric disorders among health care workers (35% of doctors and 25% of nurses) in this hospital who responded. However, doctors were more likely than nurses to suffer from a psychiatric disorder and health care workers who were single were at higher risk. Interestingly, there was no significant difference between those who were or were not exposed to SARS patients and those working in high-risk areas or those in the general wards. This study showed that there was a significant increase in the prevalence of PTSD. As the survey was conducted 2 months after the outbreak of SARS, the IES scores suggested that approximately 20% of the doctors and nurses were suffering from PTSD. In Singapore, all suspect or probable cases of SARS are transferred immediately upon diagnosis to the SARS-designated hospital. The hospital in this study was not the SARS- designated hospital and only a small number of SARS patients were identified here. Perhaps this limited contact with SARS patients in the study hospital may have resulted in this unexpected outcome. |
| Chen (2005) | Taiwan | SARS | PTSD, Depression | After | Cross-sectional | Self-reported questionnaire | 128 | Impact of Event Scale (IES)  90-item Symptoms Checklist Revised | The results revealed that characteristics of SARS-related stress reaction syndrome included anxiety, depression, hostility, and somatization. Eleven percent of the nurses were found to have stress reaction syndrome, the highest rate of which was found in the group originally working in a higher-risk unit. PTSD was variably affected by personal coping strategies, individual vulnerability, a buffer effect of training and experience, and nurses’ willingness to provide care. High level of training, professional experience, and good social support might have buffered the average severity of symptoms. |
| Chen (2006) | Taiwan | SARS | Depression, Anxiety | During and after | Prospective cohort | Self-reported questionnaire | 116 | Zung’s self-rating anxiety scale (SAS)  Zung’s self-rating depression scale (SDS)  Pittsburgh sleep quality index (PSQI) | A lower perception of work stress before SARS was associated with a higher anxiety and depression level. Those who did not volunteer to care for SARS patients had a higher anxiety and depression level. Greater family support was associated with lower depression levels, and better sleep quality. Belief that current protective equipment is sufficient is associated with lower depression levels. |
| Chen (2007) | Taiwan | SARS | PTSD | During | Cross-sectional | Self-reported questionnaire | 90 SARS HCWs; 82 control subjects | Medical Outcomes Study (MOS) - 36-Item Short Form Health Survey (SF-36) | Healthcare workers' mental health were poorer compared to the general population. Contact-days, contact-hours per day, contact-hours with symptomatic patients with SARS had significant correlation with PTSD domains. |
| Cheng* (2004) | Hong Kong | SARS | Depression, Anxiety | After | Cross-sectional | Self-reported questionnaire | 20 | General Health Questionnaire (GHQ-28) | HCWs had worse adjustment outcomes such as quality of life, distress, and self-esteem than non-HCWs. Compared with community patients, HCWs were found to have a more negative appraisal of SARS impact in the acute phase (Cheng et al., in press). Recovered HCWs might encounter prominent worries and anticipatory anxiety in returning to the workplace where they had such traumatic experiences. Thus, associated fear, avoidance, and distress related to resuming duties may be particularly salient in the initial stage of recovery. In addition, the low self-esteem of HCWs may be related to the change of perceived self-image from health protector to virus spreader. |
| Chong (2004) | Taiwan | SARS | Anxiety, depression, PTSD | During | Cross-sectional | Self-reported questionnaire | 1257 | Impact of Event Scale (IES)  Chinese Health Questionnaire (CHQ-12) | Significantly higher IES scores were shown in men, technicians, those with work experience of less than 2 years, during the repair phase (when the infection was being brought under control), among those exposed to SARS and those not living with their family. Significantly higher risk of psychiatric morbidity as assessed by CHQ-12 is associated with exposure to SARS and during the repair phase of the epidemic. |
| Chua (2004) | Hong Kong | SARS | Psychological distress | During | Cross-sectional | Self-reported questionnaire | 271 HCWs;  342 healthy control subjects | Perceived Stress Scale (PSS-10) | HCWs appeared to be protected from stress, with significantly more positive psychological effects than were observed in control subjects. HCWs who were confident about infection control had lower stress levels and fewer negative psychological effects. |
| Fiksenbaum (2006) | Canada | SARS | Psychological distress | During | Cross-sectional | Self-reported questionnaire | 333 | Emotional exhaustion subscale of the Maslach Burnout Inventory | Lower levels of organisational support, contact with SARS patients, higher levels of perceived SARS threat, time spent in quarantine were associated with higher levels of emotional exhaustion. |
| Goulia et al (2010) | Greece | Influenza A/H1N1 | Anxiety, Psychological distress | During | Cross-sectional | Self-reported questionnaire | 469 | General Health Questionnaire (GHQ-28) | More nurses and medical staff scored >5 GHQ-28 compared to allied and non-patient personnel. Degree of worry independently associated with psychological distress levels. More than half of HCWs experienced moderately high levels of worry about the pandemic, with auxiliary staff being more worried than all other groups and nurses being more worried than medical staff. Frequent concerns include risk of infection to family and friends, worries about dangerousness of disease, consequences on their functional ability. Medical staff were at least worried possibly because they mostly regarded themselves as sufficiently informed. |
| Ho (2005) | Hong Kong | SARS | PTSD | After | Cross-sectional | Self-reported questionnaire | 97 | Chinese Impact of Event Scale (CIES) | Most prevalent contributing factors of PTSD were accidental transmission of infection to family members and friends, depriving workers of social support. |
| Jung (2020) | South Korea | MERS | PTSD | After | Cross-sectional | Self-reported questionnaire | 147 | Impact of Event Scale – Revised Korean version (IES)  General Health Questionnaire (GHQ-12) | Department (highest in ICU), mental health (higher GHQ score), and level of involvement during MERS outbreak (directly involved with infected patients vs suspected) were associated with PTSD. Mental health (GHQ score lower) was negatively associated with supervisor support. |
| Kang (2020) | China | COVID-19 | Anxiety | During | Cross-sectional | Self-reported questionnaire | 994 | Patient Health Questionnaire-9 (PHQ-9)  General Anxiety Disorder-7 criteria (GAD-7)  Insomnia Severity Index (ISI-7) | 36.3% had received psychological materials, 50.4% had obtained psychological resources available through media, and 17.5% had participated in group psychological counseling. Those with severe disturbances had accessed fewer psychological materials and psychological resources available through the media. Medical and nursing staff with subthreshold disturbances most wanted to obtain skills to help alleviate others’ psychological distress, whereas other medical and nursing staff most wanted to obtain self-help skills. Medical and nursing staff with higher levels of mental health problems were more interested in skills for self-rescue and showed more urgent desires to seek help from psychotherapists and psychiatrists. |
| Khalid (2016) | Saudi Arabia | MERS | Anxiety, psychological distress | During | Cross-sectional | Self-reported questionnaire | 117 | The study tool was a comprehensive questionnaire derived and modified from the one used by Lee et. al. (2010) for the hospital staff during the 2003 SARS epidemic.  We termed it “MERS-CoV staff questionnaire”. | Contributing factors for morbidity include seeing colleagues contracting the infection, getting sicker, and being intubated for respiratory failure, and caring for these sick colleagues also put them under enormous emotional burden. Symptoms were alleviated by positive attitude in the workplace was the biggest impact in reducing staff stress, infected colleagues getting better, adequate provision of protective equipment, drop in disease transmission after strict infection control practices eased the anxiety of the staff. |
| Koh (2005) | Singapore | SARS | PTSD | During | Cross-sectional | Self-reported questionnaire | 10511 | Impact of Event Scale (IES) | Those with higher rates of PTSD perceived a greater risk of exposure to SARS because of occupational obligations, social isolation, increased stress. PTSD was alleviated by preventive measures that were effective, use of personal protective equipment, and accurate information on SARS provided. |
| Lai (2020) | China | COVID-19 | PTSD, anxiety | During | Cross-sectional | Self-reported questionnaire | 1257 | Patient Health Questionnaire-9 (PHQ-9)  Insomnia Severity Index (ISI-7)  General Anxiety Disorder-7 criteria (GAD-7) | More severe symptoms in all areas: nurses, women, frontline workers. Significantly higher symptoms of depression, anxiety, insomnia, and psychological distress in front-line workers, compared to the second-line workers. |
| Lancee (2008) | Canada | SARS | Depression, Anxiety, Substance misuse | After | Cross-sectional | Self-reported questionnaire and structured interviews | 139 | Impact of Event Scale (IES)  Kessler Psychological Distress Scale  Emotional exhaustion scale of the Maslach Burnout Inventory (MBI-EE) | Significant association of the onset of any axis I diagnosis after SARS with a previous psychiatric history. Inverse association of the onset of any axis I diagnosis after SARS with years of health care experience and the perception of being adequately trained and supported by the hospital or clinic. |
| Lee* (2018) | South Korea | MERS | PTSD | During and after | Prospective cohort | Self-reported questionnaire | 1st survey = 359  2nd survey = 77 | Impact of Event Scale – Revised Korean version (IES) | The IES-R identified 42 respondents (54.5%) as having the presence of PTSD-like symptoms, and 31(40.3%) as being eligible for a diagnosis of PTSD. Those who performed MERS-related tasks (vs those who did not) had higher mean IES-R score, higher total IES-R score, and higher IES-R subscores. |
| Liang (2020) | China | COVID-19 | Depression and anxiety | During | Cross-sectional | Self-reported questionnaire | 59 | Zung’s self-rating anxiety scale (SAS)  Zung’s self-rating depression scale (SDS) | Zung's self-rating depression scale showed higher rates of depression in COVID healthcare workers above 30 years old. Zung's self-rating anxiety scale showed no higher rates of anxiety than in other departments. |
| Lin (2007) | Taiwan | SARS | PTSD | After | Cross-sectional | Self-reported questionnaire | 83 valid DTS-C reports;  90 valid CHQ-12 reports. | Davidson Trauma Scale (DTS-C)  Chinese Health Questionnaire-12 (CHQ-12) | The average DTS-C scores of staff in the emergency department were significantly higher than those of staff in the psychiatric ward. Staff in the emergency department experienced the PTSD symptoms ‘‘acting and feeling as if the trauma were recurring’’ and ‘‘irritability’’ more severely and more often than staff in the psychiatric ward. Staff in the emergency department experienced more difficulty in ‘‘getting along with the family or friends’’ than did staff in the psychiatric ward. Self-observation about the severity of the stress caused by SARS was the only factor that was significantly different in DTS-C > 40 and DTS-C < 40. |
| Liu (2012) | China | Influenza A/H1N1 | Depression | After | Cross-sectional | Self-reported questionnaire | 549 | Center for Epidemiologic Studies Depression Scale (CES-D) | Our results support previous research indicating that hospital staff and other individuals who spend time in quarantine because of an infectious disease outbreak, may be at elevated risk for depression, even over the long term. It is likely that the relatively high post-outbreak levels of depressive symptoms found among the quarantined respondents in the current study resulted from several factors associated with quarantining, including the elevated sense of danger associated with being quarantined, reduced social support, and increased stigmatization. Pre-outbreak traumatic experience was also found, in our study, to be predictive of post-outbreak level of depressive symptoms. Of the 4 trauma exposure measures, representing exposures either to the outbreak or to other potentially traumatic experiences, we found that 3, that is, having worked in locations where exposure to patients with SARS was common, having been quarantined during the outbreak, and having been exposed to a violent incident or disaster before the outbreak, were significantly predictive of current level of depressive symptoms. A relative or friend having contracted SARS, on the other hand, was not found to be predictive of a respondent's current level of depressive symptoms. |
| Lu (2006) | Taiwan | SARS | Depression, Anxiety | During | Cross-sectional | Self-reported questionnaire | 127 | Eysenck Personality Questionnaire (EPQ)  Chinese Health Questionnaire (CHQ) | Factors associated with worse mental health after experiencing a disaster included lack of maternal caring , being overprotected by the mother and higher levels of neuroticism. Maternal protection affected the participants’ neuroticism positively and directly, and that neuroticism showed a positive and direct influence on mental symptoms, which meant that maternal protection showed an indirect influence on the mental symptoms of the participants. Nurses had less psychological morbidity. |
| Lung (2009) | China | SARS | Psychological distress | After | Prospective cohort | Self-reported questionnaire | 127 | Chinese Health Questionnaire (CHQ) | Mental symptoms were associated with daily-life stress and not the SARS crisis. Physicians had more somatic symptoms than nurses. |
| Matsuishi (2012) | Japan | Influenza A/H1N1 | PTSD | During | Cross-sectional | Self-reported questionnaire | 1625 | Impact of Event Scale (IES) | With regard to jobs, nurses and others were significantly more anxious about infection. ‘Exhaustion’ and ‘workload’ were significantly stronger in nurses than in MDs. Moreover the total IES score was significantly higher in nurses and others than in MDs. Hospital workers in high-risk work environments felt significantly more ‘anxiety about infection’, ‘exhaustion’ and ‘workload’ and had significantly higher total IES scores than workers in low-risk work environments. |
| Maunder (2006) | Canada | SARS | Burnout  Psychological distress  PTSD | After | Cross-sectional | Self-reported questionnaire | 774 | Impact of Event Scale (IES)  Kessler Psychological Distress Scale  Emotional exhaustion scale of the Maslach Burnout Inventory (MBI-EE) | Prevalence of the following functional indicators of distress since the SARS outbreak was higher in Toronto HCWs:  - decrease in patient contact and work hours,  - increase in substance use and other traits that interfere with function,  - more days off work  Maladaptive coping and perceived adequacy of training together with protection and support  - explained some of the variance in burnout  - explained some of the variance in posttraumatic stress  Maladaptive coping and attachment anxiety, together with a protective effect of experience in healthcare  - explained 31% of the variance in psychological distress.  Multiple adverse outcomes is associated with  - Longer duration of perceived risk |
| McAlonan (2007) | Hong Kong | SARS | Perceived stress,  Depression,  Anxiety,  PTSD | During and after | Prospective cohort | Self-reported questionnaire | 2003 survey = 176  2004 survey = 184 | Perceived Stress Scale (PSS-10)  Impact of Event Scale (IES)  Depression, Anxiety and Stress Scale - 21 Items (DASS-21) | In 2003, Hong Kong HCWs in study have mean PSS-10 scores higher than the normative value in a US community sample. High-risk HCWs (compared to low risk HCWs) selected had a significantly higher percentage of negative responses and a higher percentage reported fatigue, poor sleep, worry about health, and fear of social contact.  In 2004 (1 year post-epidemic), low-risk health care workers PSS score had general trend toward a decrease over time while high-risk health care workers PSS score had general trend toward an increase over time  Among high-risk health care workers, PSS was significantly higher among men. In low-risk health care workers, PSS was significantly correlated with age  PSS scores were significantly and positively correlated with DASS-21 Depression subscale scores, DASS-21 Anxiety subscale scores, and DASS-21 total scores.  Hence perceived stress was associated with higher levels of depression, anxiety and general psychological distress. |
| Mishra (2016) | India | Influenza A/H1N1 | Anxiety | During | Cross-sectional | Self-reported questionnaire | 271 | Beck's Anxiety Inventory | All medical and dental staff reported mostly low levels of anxiety. |
| Nickell (2004) | Canada | SARS | Psychological distress | During | Cross-sectional | Self-reported questionnaire | 510 | General Health Questionnaire (GHQ-12) | Emotional distress seen in nurses (45.1%) and allied health care professionals (33.3%). Lower scores seen in doctors (17.4%) and non-patient care staff (18.9%)  Part-time employment status, lifestyle affected by SARS outbreak, ability to do one's job because of precautionary measures associated with higher emotional distress. |
| Park (2008) | South Korea | MERS | Psychological distress | During | Cross-sectional | Self-reported questionnaire | 187 | 36-Item Short Form Health Survey (SF-36)  Perceived Stress Scale (PSS-10) | Poorer SF-36 scores are associated with increased stigma, high stress and low hardiness. Increased PSS-10 scores are associated with increased stigma. |
| Phua (2005) | Singapore | SARS | PTSD | During | Cross-sectional | Self-reported questionnaire | 96 | Impact of Event Scale (IES)  General Health Questionnaire (GHQ-28) | With a supportive hospital and departmental environment, the physicians and nurses of the study ED chose adaptive coping in response to the 2003 SARS outbreak, and reported low psychiatric morbidity.  Less-useful coping strategies, stressful environment, cumbersome PPE use, strict infection control measures and knowledge of colleagues falling sick contributed to PTSD. Humour and religion alleviated symptoms. |
| Poon (2004) | Hong Kong | SARS | Anxiety | During | Cross-sectional | Self-reported questionnaire | 1926 | State-Trait Anxiety Inventory (STAI) | Score ranged from 20-80 on STAI. Higher anxiety was seen in higher contact with SARS patients, healthcare assistants, nurses. Anxiety scores correlated with burnout and discomfort from wearing protective gear. |
| Sim (2004) | Singapore | SARS | Psychological distress, PTSD | After | Cross-sectional | Self-reported questionnaire | 47 | General Health Questionnaire (GHQ)  Impact of Events Scale (IES) | No association between severe psychiatric symptoms and PTSD. No significant difference between age groups, marital status. Symptoms were alleviated by support from colleagues, taking precautionary measures, getting clear directives and disease information which helped participants cope with the psychological impact of the epidemic. |
| Son (2019) | South Korea | MERS | PTSD | After | Cross-sectional | Anonymous self-reported questionnaire | 280 | Impact of Event Scale - Revised Korean version (IES) | HCW were shown to be at higher risk than non-HCW for PTSD, associated with higher perceived risk, negative emotional experience. |
| Styra (2008) | Canada | SARS | PTSD | After | Cross-sectional | Self-reported questionnaire | 248 | Impact of Events Scale - Revised (IES-R) | Contributing to PTSD:  - High-risk unit  - Taking care of only 1 patient with SARS was more stressful than none or 2/more  - Perception of risk to themselves  - Impact of SARS crisis to work life  - Depressive affect (symptoms of avoidance, hyperarousal, intrusion) |
| Su (2007) | Taiwan | SARS | Depression,  Anxiety, PTSD | During | Prospective cohort | Self-reported questionnaire and structured interviews | 102 | Beck's Depression Inventory (BDI)  Spielberger Trait Anxiety Inventory (STAI)  Davidson Trauma Scale - Chinese Version (DTS-C)  Pittsburgh Sleep Quality Index (PSQI) | Anxiety at entry was greater in both SARS ICU and regular SARS unit groups than in CCU but not different from that of the Neurology unit subjects. No differences were observed among the four groups at the end of the study. Depressed subjects were more symptomatic than the non-depressed subjects overall during the study period and also at every follow-up time point. 100% of those with past depression had symptomatic relapse when placed in this overwhelmingly stressful environment. Previous history of mood disorders might predict the occurrence of depressive disorder (MINI) and insomnia  Post-traumatic stress symptoms increased during the outbreak in nurses not only at the SARS units but also outside the SARS unit with uncertainty for displacement. Three unit subjects (SARS ICU, SARS regular, and Neurology) had higher rate of symptomatic PTSD than those of CCU.  SARS unit nurses had higher DTS-C score for the 4-week period than non-SARS unit nurses. However, there was no time and group interaction effect, which implied the same magnitude of decrease for both SARS and non-SARS group subjects.  Perceived negative feelings towards SARS at baseline was highly associated with symptomatic PTSD and insomnia**.** The rate of insomnia was fourfold higher among SARS unit nurses than non-SARS unit nurses  These findings showed that, at the end of study, CCU nurses had the least anxiety and lowest insomnia rate while Neurology unit nurses became more anxious and sleepless. Insomnia rate was two- to threefold greater in the depressed than non-depressed subjects  Depression symptom ratings decreased as the SARS epidemic decreased (with time) regardless of which group. The time by group interaction effect was reflected mainly by greater reduction of anxiety in both SARS unit groups (12–17%), in contrast to no change in the Neurology group and slight decrease in anxiety score (6%) in the CCU group. Positive attitude towards SARS was associated with decreasing depression. PTSD symptom scores decreased by 50% at the end of the study for each group. |
| Tam (2004) | Hong Kong | SARS | Emotional Distress | After | Cross-sectional | Self-reported questionnaire | 648 | General Health Questionnaire (GHQ-12) | Female, poor self-rated physical health, high level of job-related stress, inadequate support ((i) counseling and psychological support from employer and (ii) insurance and compensation) were associated with higher risk of PTSD. |
| Tang (2017) | China | Influenza A/H7N9 | PTSD  Anxiety  Depression | During | Cross-sectional | Self-reported questionnaire | 102 | PTSD Checklist – Civilian Version (PCL-C) | Factors associated with higher PCL-C scores:  - Female  - Age between 20 and 30 years  - Participants with low professional titles and high contact frequency  - Staff members with less than five years of work experience received higher scores in all dimensions than those who were working for more than five years |
| Tham (2005) | Singapore | SARS | PTSD | After | Cross-sectional | Self-reported questionnaire | 124 | Impact of Events Scale (IES)  General Health Questionnaire (GHQ-28) | Six months after SARS, the rates of post-event and psychiatric morbidity were relatively low among the ED doctors and nurses. The results might have underestimated actual morbidity as the study was conducted six months after the outbreak. |
| Verma (2004) | Singapore | SARS | PTSD | After | Cross-sectional | Self-reported questionnaire | 1058 | General Health Questionnaire (GHQ-28)  Impact of Events Scale - Revised (IES-R) | GPs diagnosed with more cases of anxiety (GHQ-28 score 7 or more) due to higher contact time with SARS patients. Mean score of GHQ somatic, anxiety, and social dysfunction subscales were significantly higher in GPs  Contirbuting factors to morbidity:  younger, higher IES-R scores, higher stigma subscales (how HCW feel and streated during outbreak; personalised stigma, disclosure concerns, negative self-image, concern with public attitudes towards HCW), direct involvement |
| Wu (2008) | China | SARS | Alcohol abuse, PTSD, depression | After | Cross-sectional | Self-reported questionnaire | 549 | Impact of Events Scale - Revised (IES-R)  Center for Epidemiologic Studies Depression Scale (CES-D)  National Household Survey on Drug Abuse (NHSDA) | Alcohol use positively associated with quarantine, working in high-risk locations (SARS wards, during outbreak), symptoms of PTSD (particularly hyperarousal symptoms), depression, drinking as a coping method. Not affected by families or friends contracting the virus. |
| Wu (2009) | China | SARS | PTSD | After | Cross-sectional | Self-reported questionnaire | 549 | Impact of Events Scale - Revised (IES-R) | Respondents who had been quarantined, or worked in high-risk locations such as SARS wards, or had friends or close relatives who contracted SARS, were 2 to 3 times more likely to have high PTS symptom levels. Persistently high PTSS ~40% (22/55 who had high PTSS at some point) (throughout the three years) significantly associated with being single, low household income. |
| Xiao (2020) | China | COVID-19 | Anxiety | During | Cross-sectional | Self-reported questionnaire | 180 | Self-Rating Anxiety Scale (SAS)  Pittsburgh Sleep Quality Index (PSQI) | Higher level of anxiety led to poorer outcomes; higher level of social support led to better sleep quality, lower anxiety led to better outcomes. |

*Repeated article

**Table B.3 Summaries of studies (n = 26) for mental health impact on patients and quarantined individuals**

| **Author (year)** | **Country** | **Subgroup** | **Respiratory epidemics** | **Reported outcome** | **Timeframe** | **Study design** | **Data collection** | **Sample size** | **Scale used** | **Results** |
| --- | --- | --- | --- | --- | --- | --- | --- | --- | --- | --- |
| Bonanno (2008) | Hong Kong | Patients | SARS | Psychological distress | After | Prospective cohort | Interview | First interview = 951  Second interview = 977  Third interview = 856 | 12-item Medical Outcome Study Short-Form Health Survey (SF-12). | Means for the MCS and PCS were below population norms at each  assessment.  Predictors of chronic dysfunction trajectory included: poor physical health, low levels of social support, high levels of SARS-related worry.  Predictor of resilience trajectory: male gender. |
| Chen (2017) | China | Patients | Influenza A/H7N9 | Psychological distress | After | Prospective cohort | Interview (face-to-face or telephone) | Months after discharge:  3 months = 37  6 months = 37  12 months = 41  24 months = 20 | 36-Item Short Form Health Survey (SF-36) | The scores for all domains of the SF-36 did not change significantly from 3 to 24 months after discharge from the hospital.  The scores for role-physical (RP) and role-emotional (RE) domains were significantly lower than those of the control population during the first year.  RP remained lower than that of the controls, but there was no difference in RE at the 24-month follow-up.  Social functioning (SF) and body pain (BP) were both lower than those of the controls; a significant difference was detected in the former at the 6-month follow-up and in the latter at the 12- and 24-month follow-up visits.  Generally, patients with no ARDS reported higher scores on all the domains of quality of life except for RE, which were comparable between patients with ARDS and patients without ARDS across the study period. |
| Cheng (2004)^α^ | Hong Kong | Patients | SARS | Psychological distress,  Depression, Anxiety | After | Cross-sectional | Self-reported questionnaire | 100 | General Health Questionnaire-28 (GHQ-28) | SARS survivors tended to be worse in most of the subscales in QOL and psychological distress compared with the community counterparts.  Amongst SARS survivors, factors associated with higher levels of psychological distress were: healthcare workers, severity of SARS symptoms, average daily steroid dosage in acute phase. |
| Cheng (2004)^β^ | Hong Kong | Patients | SARS | Depression, Anxiety | After | Cross-sectional | Self-reported questionnaire | 180 | Beck's Anxiety Inventory (BAI),  Beck's Depression Inventory (BDI) | When compared with the local healthy community sample or with psychiatric outpatients with anxiety or depressive disorders, SARS survivors have a level of psychological distress lying between the two groups.  Factors associated with anxiety symptoms: female, healthcare workers  Factors associated with depressive symptoms: chronic disease, family member killed by SARS |
| Cheng (2006) | Hong Kong | Patients | SARS | Anxiety  Depression | During | Cross-sectional | Self-report questionnaire | 57 | Beck Depression Inventory (BDI), Beck Anxiety Inventory (BAI) | HCWs have higher anxiety and depression scores.  Females have higher anxiety scores.  Higher scores on ‘perceived impact’ were correlated with higher levels of depression and anxiety, and poorer health. |
| Chua (2004) | Hong Kong | Patients | SARS | Stress,  Psychological impact | During | Cross-sectional | Self-reported questionnaire | 224 | Perceived Stress Scale-10 (PSS-10) | Increased age correlates with increased stress for healthy control subjects only.  The single most common response among both healthy control subjects and patients was worry about health. More than 20% of healthy control subjects and patients shared worry about finances or had fear of social contact. The patients also disclosed numerous additional problems, including poor sleep, weepiness, loneliness, boredom, poor concentration, depressed mood, nightmares, and impaired judgement.  Patients who were healthcare workers had significantly more positive, as well as negative psychological effects, compared with other patients. Increased stress significantly correlated with more negative psychological effects. |
| Han (2011) | China | Patients | Influenza A/H1N1 | Narcolepsy | After | Case-control | Telephone Interview (telephone) | 906 | Stanford Sleep Inventory (SSI) | Studying year-to-year variation, Han (2011) found a 3-fold increase in narcolepsy onset following the 2009 H1N1 winter influenza pandemic.  The increase was unlikely to be explained by increased vaccination. |
| Hawryluck (2004) | Canada | Quarantined Individuals | SARS | Depression, PTSD | During | Cross-sectional | Web-based survey | 129 | Impact of Events Scale-Revised (IES-R),  Center for Epidemiologic Studies - Depression Scale (CES-D) | Factors correlated with both PTSD and depressive symptoms: lower combined annual household income, acquaintance with or exposure to someone who was hospitalized with SARS, those who wore their masks all of the time.  Factors correlated with PTSD symptoms only - Duration of quarantine  All respondents described a sense of isolation. Lack of social and, especially, the lack of any physical contact with family members were identified as particularly difficult. Confinement within the home or between work and home, not being able to see friends, not being able to shop for basic necessities of everyday life, and not being able to purchase thermometers and prescribed medications enhanced their feeling of distance from the outside world. Infection control measures imposed not only the physical discomfort of having to wear a mask but also significantly contributed to the sense of isolation. In some, self-monitoring of temperature provoked considerable anxiety. |
| Hong (2009) | China | Patients | SARS | PTSD | After | Prospective cohort | Interview (face-to-face or telephone) or Mailing | Months after discharge:  2 months = 70, 7 months = 68,  10 months = 65,  20 months = 57,  46 months = 43 | Impact of Events Scale (IES),  Zung's Self-Rating Anxiety Scale (SAS),  Zung's Self-Rating Depression Scale (SDS),  36-Item Short Form Health Survey (SF-36),  Symptom Checklist- 90 (SCL-90) | IES, SAS, SDS and SCL-90 were higher, and functional impairment as measured by SF-36 and SDSS was more severe for subjects with PTSD |
| Hui (2009) | Hong Kong | Patients | SARS | Psychological distress | After | Prospective cohort | Interview | 123, 110, 97 | 36-Item Short Form Health Survey (SF-36) | Functional disability out of proportion to degree of lung function impairment may be due to psychological factors |
| Jeong (2016) | South Korea | Quarantined individuals | MERS | Anxiety | During | Cross-sectional | Interview | 1692 | Generalized Anxiety Disorder 7-item criteria (GAD-7) | Risk factors for experiencing anxiety symptoms and anger at four to six months after release included: experiencing symptoms related to MERS during isolation, inadequate supplies (food, clothes, accommodation), social networking activities (email, text, Internet), history of psychiatric illnesses and financial loss. |
| Kim (2018) | South Korea | Patients | MERS | Stress, Depression,  PTSD | During | Cross-sectional | Medical records | 40 | Patient Health Questionnaire-9 (PHQ-9),  Impact of Events Scale-Revised (IES-R),  Peritraumatic Dissociation- Posttraumatic Negative Beliefs-Posttraumatic Social Support scale (PTD-PTNB-PTSS) | Patients with mild-to-severe depression had significantly higher mean scores on the IES-R , and PTD than those without depression.  The PHQ-9 scores were significantly positively correlated with the IES-R, PTD, and PTNB scores.  Multiple linear regression analysis revealed that only the IES-R scores were significantly positively correlated with PHQ-9 scores. |
| Kwek (2006) | Singapore | Patients | SARS | Anxiety,  Depression, PTSD | During | Cross-sectional | Self-reported questionnaire | 63 | Hospital Anxiety and Depression Scale (HADS),  Impact of Events Scale (IES),  36-Item Short Form Health Survey (SF-36) | ICU patients suffered more in terms of role restriction due to physical problems. They also had a worse vitality score. |
| Lam (2009) | Hong Kong | Patients | SARS | Anxiety, Depression, PTSD, Chronic fatigue syndrome | After | Prospective cohort | Interview | 233 | Hospital Anxiety and Depression Scale (HADS),  Modified criteria for chronic fatigue syndrome (CFS) according to the Centers for Disease Control and Prevention (CDC),  Chinese version of the Chalder Fatigue Questionnaire | Factors associated with increased risk of poorer mental health outcomes at follow-up included: being a healthcare worker, being unemployed (including housewives and retirees), perception of social stigmatization and having applied to SARS survivor's fund.  Shorter duration of follow-up study and application to SARS survivor's fund was associated with CFS in SARS survivors.  Those with psychiatric morbidities have a higher percentage of individuals having no gainful employment at follow-up. |
| Lee (2004) | Hong Kong | Patients | SARS | Psychosis | During | Case-control | Medical records | 15 | DSM-IV criteria | Family history of psychiatric illness was significantly more common among patients with SARS-related psychosis than among control subjects.  Among patients with SARS-related psychosis, there was also a higher rate of family members contracting SARS, but the trend was marginally insignificant.  All case patients and control subjects had received steroid treatment for SARS. The patients with SARS-related psychosis received significantly higher cumulative doses of steroids during their inpatient hospital stays than did the control subjects. |
| Lee (2007) | Hong Kong | Patients | SARS | Stress, Depression, Anxiety, PTSD | During | Prospective cohort | Self-reported questionnaire | 79 | Perceived Stress Scale-10 (PSS-10) | Mean PSS-10 scores of SARS survivors were significantly higher than those of matched community control subjects in 2003.  At both time points, PSS-10 scores were higher among female survivors.  Mean PSS-10 scores of SARS survivors did not decrease between 2003 and 2004.  During the outbreak, healthcare worker and non–healthcare worker SARS survivors had similar PSS-10 scores |
|  |  |  |  |  | After |  |  | 96 | Perceived Stress Scale-10 (PSS-10),  Depression, Anxiety and Stress Scale - 21 Items (DASS-21),  Impact of Events Scale - Revised (IES-R),  General Health Questionnaire (GHQ-12) | Female gender and healthcare workers associated with higher PSS-10, DASS-21, IES-R and GHQ-12 scores.  When measured as continuous variables, all the measures of DASS-21, IES-R and GHQ-12 scores were strongly intercorrelated. Perceived stress (PSS) was also positively and significantly associated with DASS-21, IES-R and GHQ-12 scores.  One year after the outbreak, healthcare worker SARS survivors had significantly higher PSS-10 scores, compared with their non–healthcare worker counterparts.  The PSS-10 scores of healthcare worker SARS survivors increased significantly from the outbreak to 1 year after the outbreak, while those of non-healthcare worker SARS survivors remained similar over the period.  One year after the outbreak, healthcare worker SARS survivors were also substantially more distressed on all dimensions of psychological distress than their non–health care worker counterparts.  Even after controlling for age, sex, and education level, healthcare worker status is associated with a six- fold increased chance for GHQ-12 caseness. |
| Cho (2020) | South Korea | Quarantined Individuals | MERS | PTSD | After | Retrospective cohort | Self-reported questionnaire | 67 | Impact of Events Scale-Revised-Korean Version (IES-R-K) | Women and patients who had a shorter duration of isolation were more likely to develop symptoms of IES-R-K≧18.  The sleep disturbance subscale score was higher among females, compared to male subjects.  Participants isolated for less than 16 days showed:  - A higher total score than those isolated for more than 16 days,  - At a subscale level, avoidance and sleep disturbance, emotional numbing, and dissociation subscale scores were higher (in particular: emotional numbing and dissociation scores). |
| Lee (2019) | South Korea | Patients | MERS | Chronic fatigue,  Depression,  PTSD | After | Prospective cohort | Self-reported questionnaire | 52 | Impact of Events Scale-Revised (IES-R),  Patient Health Questionnaire-9 (PHQ-9),  Fatigue Severity Scale (FSS). | With T1 referring to 12 months after the epidemic, and T2 referring to 18 months after the epidemic.  Mean scores of all of the scales were lower at T2 than at T1  FSS (T1) scores were positively correlated with scores on the PHQ-9 (T1, T2) and the IES-R (T2).  T1 PHQ-9 was associated with T2 PHQ-9.  T2 PHQ-9 was associated with T2 IES-R. |
| Tansey (2007) | Canada | Patients | SARS | Psychological distress | After | Prospective cohort | Self-reported questionnaire | 117 | 36-Item Short Form Health Survey (SF-36) | All SF-36 domains were significantly reduced at 3 months with the exception of bodily pain.  Role physical, social function, and role emotional domains were improved at 1 year after hospital discharge but did not normalize. |
| Mak (2010) | Hong Kong | Patients | SARS | PTSD | After | Prospective cohort | Self-reported questionnaire | 90 | Structured Clinical Interview for DSM-IV (SCID) Chinese version | Factors associated with PTSD were: female, healthcare workers, pre-SARS chronic medical illness, AVN as a complication (explained by functional impairment), higher average pain in past month, higher functional impairment checklist scores, higher perceived inadequacy of social support, higher subjective perception of danger during SARS outbreak and higher subjective perception of being stigmatised during the SARS outbreak. |
| Mak IWC (2009) | Hong Kong | Patients | SARS | PTSD, Depression | After | Prospective cohort | Self-reported questionnaire | 90 | Chinese Impact of Events Scale-Revised (IES-R),  Hospital Anxiety and Depression Scale (HADS),  36-Item Short Form Health Survey (SF-36),  Structured Clinical Interview for DSM-IV (SCID) | Being a healthcare worker was associated with having chronic PTSD |
| Mak WWS (2009) | Hong Kong | Patients | SARS | Psychological distress | After | Cross-sectional | Interview | 143 | 36-Item Short Form Health Survey (SF-36) | Lower levels of psychological distress was associated with: higher education,  support from medical staff and family/friends, self-care self-efficacy. |
| Mihashi (2009) | China | Quarantined Individuals | SARS | Psychological distress | After | Cross-sectional | Self-reported questionnaire | 187 | General Health Questionnaire-30 (GHQ-30) | Factors associated with psychological distress: male gender, company/university staff occupation, positive for fever, positive for headache, restricted range of activities, restricted television viewing, restricted smoking, restricted eating, not wearing a mask, restricted going out, no continuation of schooling/working and experienced income reduction. |
| Reynolds (2008) | Canada | Quarantined Individuals | SARS | PTSD | During | Cross-sectional | Self-reported questionnaire | 1057 | Impact of Events Scale-Revised (IES-R) | Factors associated with higher IES-R score: healthcare worker status, self-reported longer duration of quarantine, compliance with SARS quarantine requirements and increasing perceived difficulty with compliance with SARS quarantine requirements. |
| Wang (2011) | China | Quarantined Individuals | Influenza A/H1N1 | PTSD | During | Cross-sectional | Self-reported questionnaire | 176 | Impact of Events Scale-Revised (IES-R),  Self-Report Questionnaire (SRQ-20) | No immediate negative psychological effect of quarantine in the sample. Significantly lower total scores of IES-R in the quarantined female group than in the non-quarantined female group.  Perceived health hazards and dissatisfaction with control measures were the significant predictors of IES-R positive screening.  Dissatisfaction with control measures significantly associated with SRQ-20 positive screen. |
| Wu (2005) | Hong Kong | Patients | SARS | Depression, Anxiety, PTSD | During | Cross-sectional | Self-reported questionnaire | 195 | Impact of Events Scale-Revised (IES-R),  Hospital Anxiety and Depression Scale (HADS) | Perceived life threat was the most significant predictor for the IES-R and HADS scores that reflect anxiety-based symptoms.  Perceived emotional support was the best predictor of the HADS score that reflects depression.  Gender and education level were found to be significantly associated with the avoidance symptoms.  Knowing someone who had SARS was significantly associated with the measure related to depression. |

^α^ Adjustment outcomes in Chinese patients following one-month recovery from severe acute respiratory syndrome in Hong Kong

^β^ Psychological distress and negative appraisals in survivors of severe acute respiratory syndrome (SARS)
